# Those That Remain: Sorption/Desorption Behaviour and Kinetics of the Neonicotinoids Still in Use

**DOI:** 10.3390/ijms24076548

**Published:** 2023-03-31

**Authors:** Gordana Sinčić Modrić, Mirna Petković Didović, Igor Dubrović, Paula Žurga, Dalibor Broznić

**Affiliations:** 1 Department of Environmental Health, Teaching Institute of Public Health of Primorje-Gorski Kotar County, Krešimirova 52a, 51000 Rijeka, Croatia; 2 Department for Medical Chemistry, Biochemistry and Clinical Chemistry, Faculty of Medicine, University of Rijeka, Brace Branchetta 20, 51000 Rijeka, Croatia

**Keywords:** acetamiprid, thiacloprid, neonics, sorption kinetics, soil

## Abstract

In January 2023, the derogation loophole was closed on “emergency authorisations” for the use of three out of five neonicotinoids in all EU states. In this study, we analysed the sorption/desorption behaviour and kinetic parameters of acetamiprid and thiacloprid, the two neonicotinoids that are still approved for use, either regularly or under emergency authorisations in the EU, and widely used worldwide. Sorption and desorption curves in four soils with different organic matter content were analysed using four kinetic models, namely, Lagergren’s pseudo first-order model, two-site model (TSM), Weber–Morris intraparticle diffusion model and Elovich’s model. Kinetic parameters were correlated to soil physico-chemical characteristics. To determine the mutual influence of soil characteristics and sorption/desorption parameters in the analysed soils, a factor analysis based on principal component analysis (PCA) was performed. Even though the two insecticides are very similar in size and chemical structure, the results showed different sorption/desorption kinetics. The model that best fits the experimental data was TSM. Thiacloprid showed a more rapid sorption compared to acetamiprid, and, in all soils, a higher proportion sorbed at equilibrium. Intra-particle diffusion seemed to be a relevant process in acetamiprid sorption, but not for thiacloprid. Desorption results showed that acetamiprid is more easily and more thoroughly desorbed than thiacloprid, in all soils. The kinetic behaviour differences stem from variations in molecular structure, causing disparate water solubility, lipophilicity, and acid–base properties.

## 1. Introduction

Nowadays, global agriculture has emerged as one of the most important branches of industry, representing the basis of the development of each country. However, modern agriculture tends to utilize all the constituents of the soil, considering only economic profitability, without maintaining the quality of arable soil [1,2,3]. The consequence of this is an increase in agricultural yields, but it is often closely related to the application of enormous amounts of chemical compounds (herbicides, fungicides, and insecticides). Neonicotinoids (or neonics) are a group of relatively recently synthesized insecticides, which have demonstrated great success in showing strong insecticidal activity and a wide spectrum of action, with minimal toxic effect on the environment and human population [4,5]. Imidacloprid was the first in use [6], becoming, in 2001, one of the best-selling insecticides in the world. After imidacloprid, the second neonicotinoid generation was synthesized, followed by acetamiprid and thiacloprid as third-generation representatives.

However, the toxicity of some of the members of the neonics family to non-target organisms, both vertebrates and mammals, is now well established [7,8,9,10,11,12,13]. Imidacloprid, thiamethoxam and clothianidin were first banned in 2013 for use on agricultural crops attractive to bees, but with the possibility of application on other crops; in 2018 they were banned for use in the EU except in greenhouses, but with were still available to use by invoking emergency authorisations [14]; finally, in January 2023, the EU’s highest court prohibited member states from issuing such authorisations [15]. This derogation loophole was thus closed for three out of five neonics; thiacloprid was not included in the ruling, while acetamiprid was and still is regularly approved in all EU states [16]. Both are also still in use around the globe [17,18,19,20,21,22,23,24,25]. The worldwide study of neonics in honey found that 75% of honey samples contained at least one neonic, with acetamiprid and thiacloprid showing the highest maximum and average concentrations among positive samples [26]. It is likely that their consumption will further increase in following years, since the banned neonics are likely to be replaced with those ones still allowed [22]. This scenario also includes Croatia, an EU member state, where neonics are applied to approximately 30% of agricultural land [27,28].

It is well known that, from the total neonicotinoid active substance applied in seed treatment, only 2–20% is sorbed by the crop, while the rest is transferred on or through the soil ecosystem [12,13,29] (Note that application rates are up to 0.62 kg and 0.57 kg active ingredient per hectare for acetamiprid and thiacloprid, respectively [30,31]). The fate and distribution of those active substances are controlled by the sorption, desorption and degradation processes, which, in turn, depend on soil physico-chemical properties [19,32,33,34,35,36]. Generally, soil degradation is faster for acetamiprid, with a half-life in the field of 3 days, compared to 8.8 days for thiacloprid [17]. The efficiency of sorption and an insight into sorption mechanism is gained through the use of sorption isotherms, i.e., the plots of the amount of pesticide sorbed by soil versus equilibrium pesticide concentration in the solution. The efficiency of sorption is quantified by a constant *K*_d_ when the isotherms are linear, or *K*_f_ when the isotherms are described by the conventional Freundlich model. The parameter *K*_OC_ is obtained when respective sorption constants are normalized to organic carbon content (OC), *K*_OC_ = *K*/OC. The use of this parameter is justified when the sorption is taking place exclusively in the soil organic fraction, which is assumed to have the same thermodynamic characteristics in different soils, and the sorption isotherms are linear [37]. In the Freundlich model, the parameter 1/n is also obtained, which is used to assess the heterogeneity of sorption site energies. In the present literature, there are studies reporting both linear and non-linear sorption isotherms for acetamiprid and thiacloprid. USEPA fact sheets report values of *K*_d_ < 4.1 mL/g and 1/n between 0.82 and 0.91 for acetamiprid, while equivalent data cannot be found for thiacloprid [30,31]. Oliver et al. [38] reported thiacloprid *K*_d_ values ranging from 4.6 to 35.9 mL/g, while *K*_f_ values of 1.16–9.06 mL/g were reported by Zhang et al. [39]. PPDB lists *K*_f_ values 0.6–3.13 mL/g and 1.14–5.76 mL/g for acetamiprid and thiacloprid, respectively [17]. The respective 1/n values are 0.807–0.825 and 0.833–0.914. EFSA pesticide risk assessment documents report *K*_FOC_ values of 71–138 mL/g and 393–870 mL/g for acetamiprid and thiacloprid, respectively [40,41]. A recent study of Li et al. [23] reported linear isotherms for both acetamiprid and thiacloprid, with *K*_d_ values 0.71–3.02 mL/g and 1.42–4.21 mL/g, respectively, depending on the soil depth, i.e., organic carbon content. All these values consistently show higher sorption efficiency for thiacloprid compared to acetamiprid. The differences in 1/n values indicate greater heterogeneity of sorption site energies for acetamiprid, compared to thiacloprid. Regarding acid–basic properties, the literature reports p*K*_a_ = 0.7 for acetamiprid, while thiacloprid is reported not to dissociate [17,30,31,42].

While the data regarding sorption efficiency of acetamiprid and thiacloprid (and their degradation products) are well-documented, the kinetics of the process are seldom studied. When kinetic data are fitted with mathematical models, the information about the time required to achieve equilibrium, the rate and progress of the sorption/desorption reactions, as well as insights into the mechanisms of the sorption, can be obtained. Furthermore, kinetic models are used to estimate sorption/desorption parameters that can be used in process-oriented models to predict the insecticides’ uptake into crops, their leaching, transport, and runoff in the soil system, as well as their control of weeds or pests. It is also possible to assess the potential risk contamination of groundwater as a drinking water resource [21,43,44,45,46]. Compared to other insecticides, the data on the neonics’ sorption—and especially desorption—process are quite scarce. Alongside other aspects of their behaviour, the fundamental understanding of acetamiprid and thiacloprid’s kinetics has recently been recognized as an issue of tremendous relevance [47]. To that end, in this research, we studied sorption/desorption kinetics, with three main objectives: (a) to analyse the effect of the physico-chemical soil characteristics on their sorption/desorption reaction rate and determine the distribution change between the sorbed and desorbed phases over time; (b) to find the mathematical model that best describes the experimental behaviour; (c) to use the model to evaluate the possible mechanisms of sorption/desorption kinetic processes, in order to accurately assess the environmental pollution risk at sites of application.

## 2. Results

### 2.1. Evaluation of Acetamiprid and Thiacloprid Sorption and Desorption Equilibrium Time in Experimental Soils 

**Figure 1:**
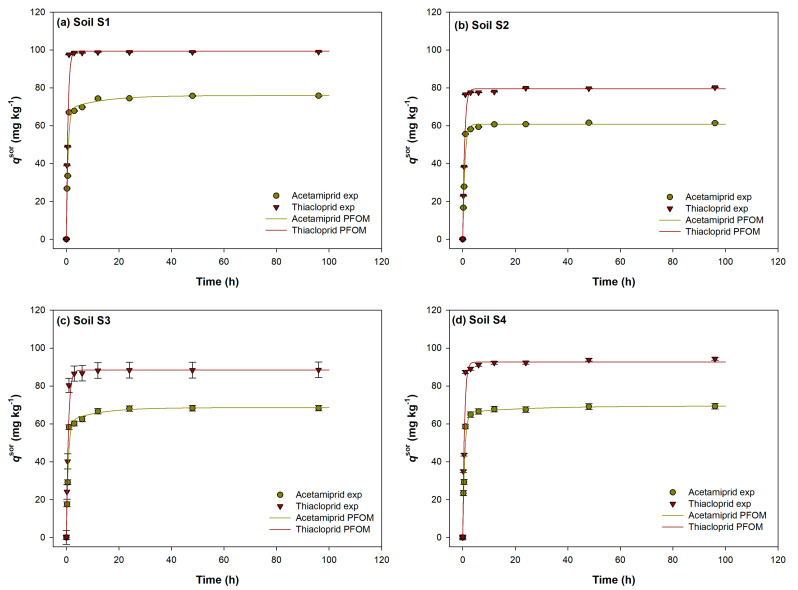
Experimental data and theoretical curves represented by nonlinear kinetics two-site model (TSM) for acetamiprid and thiacloprid sorption in experimental soils S1–S4 (**a**–**d**). Values are expressed as mean of three determinations with standard deviation.

The time taken for the sorption equilibrium to be established for both analysed neonicotinoids in soils was studied over a 96 h period, at a concentration of 30 mg/L. As can be observed in Figure 1a–d, the time needed to achieve the equilibrium was markedly longer for acetamiprid (6.5 h–47.5 h) compared to thiacloprid in all soils (thiacloprid equilibrium was reached within 9.25 h, maximally). The equilibrium time of both insecticides differed from soil to soil. For acetamiprid, the equilibrium time increased in the order: S4 (6.5 h) < S2 (18.3 h) < S3 (18.5 h) < S1 (47.5 h), while for thiacloprid, the order was somewhat different: S1 = S2 (1.8 h) < S3 (2.8 h) < S4 (9.3 h). Keeping in mind that the total organic carbon (TOC) values are the highest for soil S1, followed by S4, S3 and S2 (Table 1), it is clear there is no correlation between equilibrium time and TOC. A study of neonics’ equilibrium time in Ghanaian soils found values similar to ours for acetamiprid, but markedly longer equilibrium times for thiacloprid [21]. Likewise, thiacloprid sorption equilibrium in Mediterranean semiarid soils was reached within an interval of 24 h [48]. 

Except for the longer equilibrium times for acetamiprid, another pronounced difference between the insecticides is a higher percentage of sorption for thiacloprid in all examined soils. The acetamiprid percentage of sorption was generally low and increased in the order: soil S2 (37%) < soil S3 ≈ soil S4 (46%) < soil S1 (52%). The sorption of thiacloprid was prominently higher, but rose in the same order as for acetamiprid: soil S2 (50%) < soil S3 (59%) < soil S4 (62%) < soil S1 (66%). Comparing these trends with the TOC increase (Table 1), it is apparent that the maximum percentage of sorption is predominantly controlled by TOC. Comparable data on sorption percentages of acetamiprid and thiacloprid can be found in the literature, though with slight variations that are most often the result of differences in soil types and physico-chemical characteristics, as well as concentrations of applied insecticides. Dankyi et al. also found a stronger sorption capacity of thiacloprid (72%) compared to acetamiprid (39%) for soils in cocoa-growing regions of Ghana [21]. These thiacloprid percentages are somewhat higher compared to our study, which can be attributed to different soil characteristics (OC in the range 1.6–4.8%) and a higher application dose (200 μg/kg). Results demonstrating a higher percentage of sorption of thiacloprid, compared to acetamiprid, were also reported by Francisco et al. [49].

In all examined soils, a two-phase process was observed, consisting of an initial instantaneous rapid phase that takes place in the first few hours, depending on the insecticide, followed by a slower phase, lasting until the equilibrium time is reached. The analysed soils sorbed thiacloprid more strongly than acetamiprid, with a markedly faster initial reaction phase: in the first 15 min, an average of 45% of the applied insecticide dose of thiacloprid and 37% of the acetamiprid was already sorbed. In Mediterranean soils, Rodriguez-Liebana et al. also found significant thiacloprid sorption in the first 15 min of the reaction, up to the amount of 35% [48], while Aseperi et al. reported an extremely high percentage of sorption in UK soils (79–82%) during the rapid phase of the reaction (15 min) [33].

Furthermore, a similar trend in increasing rate continues until the first hour of the reaction, showing an even more pronounced increase in thiacloprid sorption, with an average of 56% of the total administered dose sorbed, while, in the case of acetamiprid, this percentage was evidently lower and amounted to only 40% of the administered dose. The reaction rates of the sorption process, as well as their kinetics, are discussed below.

**Figure 2:**
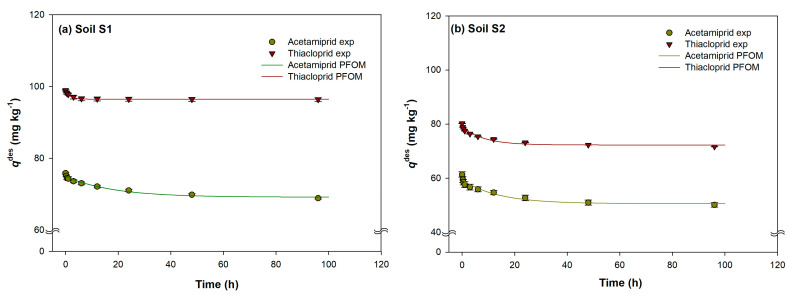

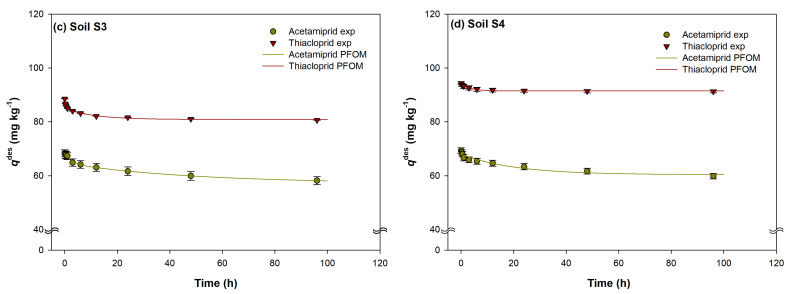
Experimental data and theoretical curves represented by two-site model (TSM) for desorption of acetamiprid and thiacloprid in experimental soils S1–S4 (**a**–**d**). Values are expressed as mean of three determinations with standard deviation.

After the sorption process, the desorption procedures were carried out, depending on the sorbed amount of each insecticide, during a 96 h time interval, and the obtained results are shown in Figure 2a–d. In the desorption process of the analysed insecticides, a longer time period was needed to reach the equilibrium conditions, compared to sorption, except for acetamiprid desorption in soil S1, where equilibrium time was shorter, with value of 23.5 h. The times needed to reach the acetamiprid desorption equilibrium also depended on the individual soil, and time increased in the order: soil S1 (23.5 h) < soil S2 (46.3 h) < soil S3 (88.0 h) < soil S4 (90.0 h). Somewhat different behaviour was obtained in thiacloprid desorption, where a shorter equilibrium time was achieved in soil S4 (26.5 h), while in the remaining soils the equilibrium process lasted significantly longer (soil S3—40.3 h; soil S2—63.3 h; soil S1—94.8 h).

As is also visible from Figure 2a–d, the retention of sorbed insecticides was observed in all studied soils. The retention was more pronounced in the case of thiacloprid desorption, where an average of 94% of the sorbed insecticide was retained, while acetamiprid was retained by the soils at an average rate of 84%. Soils S1 and S4 retained 98% and 97% of sorbed thiacloprid, respectively, while the percentages of acetamiprid sorbed in the same soils were lower, and amounted 91% and 86%. Very similar behaviour was observed in soils S2 and S3, where thiacloprid was retained at rates of 89 and 91%, respectively, and acetamiprid at 75% and 85%, respectively. In a discrepancy with our results, much lower retained values of acetamiprid (39%) and thiacloprid (72%) can be found in the literature [21].

As with the sorption process, the desorption process is also biphasic reaction, with a first rapid phase and an extended duration second phase. However, it should be noted that the biphasic nature is much less pronounced in the desorption process, and it is much slower than the sorption process. This is supported by the fact that, in the first 15 min of the desorption process, acetamiprid was desorbed on average at a rate of 3% of the sorbed insecticide, while for thiacloprid this percentage was slightly higher than 1%. It is interesting to note that, in the first 0.5 h of the process, thiacloprid was not desorbed from soil S1 (<0.02%).

The differences in the sorption kinetics of the two pesticides are in accordance with literature reports regarding their respective sorption efficiencies, namely, their sorption constants and 1/n values [17,23,40,41] (*vide supra*), and these can explain their differences. Higher values of thiacloprid sorption constants indicate stronger sorption, which is reflected in its higher percentage of sorption (Figure 1), weaker desorption, and higher retention after desorption (Figure 2). The 1/n values indicate greater heterogeneity of sorption site energies for acetamiprid, suggesting sorption that is less selective towards the soil organic matter; i.e., some sorption takes place on other soil constituents. Such data provide a plausible explanation for the longer equilibrium times of acetamiprid: while thiacloprid rapidly (and more firmly) saturates sorption sites on organic phase, without noticeable intra-particle diffusion, acetamiprid is less selective towards organic phase. After the initial fast (and less firm) saturation of organic phase sorption sites, slower intra-particle diffusion occurs, in which acetamiprid is likely sorbed to other soil constituents as well. This would be in accordance with the results of Li et al. [23], which showed a positive Kendall’s Tau correlation coefficient between acetamiprid *K*_d_ and soil CEC, but no equivalent correlation for thiacloprid. The sorption remains weaker compared to thiacloprid, resulting in easier desorption and higher desorbed amounts.

### 2.2. Sorption/Desorption Kinetic Models

To gain further insight into the mechanisms involved in the sorption and desorption processes of the analysed insecticides, the experimental data were tested by different mathematical models, and the suitability between the experimental data and those obtained by the models was assessed by the following statistical indices: coefficient of multiple determination (*R*^2^), the scaled root mean squared error (SRMSE) and the minimum error level of *χ*^2^ test (err-%).

#### 2.2.1. Pseudo-First Order Model (PFOM)

Sorption and desorption rate constants (*k*^sor^ and *k*^des^) and maximum of sorbed/desorbed amount (*q*_e_^sor^ and *q*_e_^des^) for acetamiprid and thiacloprid, as estimated by the PFO mathematical model in all soils with statistical indices, are presented in Table S1, Table 2 and Table 3. Among the applied mathematical models, PFOM gave a relatively good fit with the experimental data of the sorption processes of both applied insecticides, which is corroborated by the high *R*^2^ > 0.9961, and low error values (SRMSE = 0.0191–0.0660; err-% = 1.53–5.30). The estimated values of *q*_e_^sor^ differed very little from the experimentally obtained values (<8%) for both insecticides, and were significantly higher for the sorbed thiacloprid.

In the literature, opposite views can be found to those obtained in this study, that the estimated parameters using the PFOM do not provide a satisfactory optimization, especially when the data set of the entire time interval is modelled. It implies that, despite the high values of *R*^2^, the PFOM could be only applicable in shorter time intervals, e.g., only in the first 16 h of the thiacloprid sorption process. Furthermore, the authors indicated that the PFOM is not appropriate for determining the soil maximum sorption capacity for insecticide [48]. Azizian et al. [45], explained in their research that the use of the PFOM is justified if high initial insecticide doses are used. This fact indicates that the high dose of insecticide applied (150 mg/kg) in this study is probably the reason for the good agreement between the experimental values and those obtained from the PFOM.

#### 2.2.2. Elovich’s Model (EM)

Experimental data modelled by the EM provided a poorer explanation of the sorption processes for both insecticides than that provided by the PFOM. This was evidenced by the obtained statistical parameters, in which the fitting of experimental data by the EM generated *R*^2^ ranged from 0.6670 to 0.9880, SRMSE from 0.0437 to 0.2963, and err-% values from 3.51 to 23.79% (Table S2, Table 2 and Table 3). Furthermore, a better application of the EM in describing the acetamiprid sorption compared to thiacloprid is observed. With this biphasic model, where sorption rate is the highest at the initial phase of the process itself, after which the rate gradually decreases until the equilibrium state is reached, two parameters were estimated: (1/Y)Ln(XY)^sor^ represents the sorbed insecticide amount (mg/kg) in the rapid reaction phase (up to 1 h of reaction), while (1/Y)^sor^ is related to the coverage of the soil colloid surface with insecticide molecules, as well as to the activation energy of the chemisorption process.

Although the EM does not provide excellent agreement between the experimental and model data, its use in explaining the biphasic nature of the acetamiprid sorption process is more justified, because the fraction of insecticide sorbed during the fast reaction phase was below 90%. The values of the 1/Y^sor^ parameter for thiacloprid were lower than those obtained for acetamiprid (Table S2), as well as lower than in soils with lower TOC content, indicating a prolonged slow phase to the acetamiprid sorption process. Contrary to the sorption processes, the desorption data are very well fitted to the EM for both insecticides, meaning this kinetic model represents a very good choice for explaining the desorption processes. An excellent fit of data by EM was indicated by low errors (SRMSE < 0.0888; err-% < 7.13%) and high *R*^2^ values (>0.9449), except in the case of the desorption from soil S1, where an err-% of ≈10% was achieved (Table 2 and Table 3). The estimated values of parameters (1/Y)Ln(XY)^des^ and 1/Y^des^ obtained by the EM are listed in Table S2. For the experimental soils, the (1/Y)Ln(XY)^des^ values representing the retained amount of insecticide in the 1st h of the rapid desorption phase varied from 50 to 74 mg/kg for acetamiprid, depending on the specific soil, while for thiacloprid the values ranged from 73 to 98 mg/kg. Comparing the percentage values of insecticide retained during the rapid desorption phase, it is evident that differences between acetamiprid and thiacloprid existed. Higher percentage values were observed for thiacloprid desorption in soils S3 (44%) and S4 (37%), while, on the contrary, acetamiprid desorption was more pronounced in soils S1 and S2. Significantly lower values of the estimated parameter 1/Y^des^ were found for thiacloprid desorption (2 fold on average).

Correlation analyses of pooled sorption data for acetamiprid indicated the significant, strong, and positive correlation of (1/Y)Ln(XY)^sor^ with TOC (0.973, *p* = 0.027), with ratio 465/665 (0.998, *p* = 0.002) (Table S4). Very similar behaviour was observed for thiacloprid, with the fact that a positive and statistically significant correlation was also achieved between (1/Y)Ln(XY)^sor^ and ratio C/H (0.955, *p* = 0.045). The parameter 1/Y^sor^ for thiacloprid sorption showed a strong, negative, and statistically significant effect with ratio C/H (−0.982, *p* = 0.018), while neither correlation was statistically significant for acetamiprid. Desorption parameters (1/Y)Ln(XY)^des^ of both insecticides significantly and positively correlated with ratio 465/665 (0.980; *p* = 0.020), while, for thiacloprid, a positive correlation with TOC (0.987, *p* = 0.013) and ratio C/H (0.960, *p* = 0.040) was also observed (Table S5). The correlation revealed a negative, significantly strong influence of 1/Y^des^ for thiacloprid desorption with TOC (−0.972, 0.028) and ratio C/H (−0.999, 0.001). Acetamiprid 1/Y^des^ parameter significantly correlated with TOC (−0.959, 0.041) and ratio 465/665 (−0.996, 0.004).

Aseperi et al. ([33]), who studied the sorption of thiacloprid in the UK, determined that the EM model provided a poor description of the sorption mechanism in these soils and that the *R*^2^ values were in the range of 0.616–0.890, which is comparable to the results obtained in this study. Furthermore, in the same study, a time period of the rapid sorption phase of 6 h was determined, where the sorbed amount was 88 to 99%. Although in our study we assumed a shorter time interval of the rapid reaction phase (1 h), almost identical values were obtained, ranging from 91 to 97%. Fernandez-Bayo et al. [44], in their study of imidacloprid sorption in Spanish soils, assumed a duration of the rapid sorption phase of 1 h, in which more than 80% of the insecticide was sorbed, compared to the total amount obtained in 24 h. The aforementioned study, by Aseperi et al. [33], confirmed that the values of the sorption parameter 1/Y were lower in soils with a higher content of OM, and that the equilibrium was reached within 6 h of the sorption reaction. On the contrary, Fernandez-Bayo et al. [44], observed that the value 1/Y was lower in soils with a lower content of OM. In our study, we did not observe a correlation of 1/Y with OM, but the influence of ratio C/H on the parameter was observed in the thiacloprid sorption. The basic assumption of the EM is the heterogeneity of the soil colloid particles’ surfaces, and the significant influence of chemisorption. The EM shows the highest agreement with the experimental values for acetamiprid sorption in soil S1, indicating a significant influence of chemisorption. However, for those soils which had thiacloprid applied, the worse agreement of the experimental results and EM may be due to the fact that this model ignores the simultaneous effects of the desorption process [50].

#### 2.2.3. Intraparticle Diffusion Model (WMM)

An even weaker agreement between the experimental values and those obtained by the EM was also achieved with the application of the WM model. The values of statistical indices *R*^2^, ranging from 0.4635 to 0.9172, SRMSE, in the range from 0.1864 to 0.4025, and average value of err-% ≈ 25% indicated that this model does not provide a suitable description of the behaviour of both insecticides (Tables S3 and S5, Table 2 and Table 3).

Aseperi et al., in their research on the sorption behaviour of thiacloprid and thiamethoxam in soils with contrasting OC contents, studied the influence of intraparticle diffusion on the sorption processes, i.e., whether the sorption mechanism is controlled by the diffusion process [33]. Their results indicated the weak applicability of the WMM model, as evidenced by the low values of the parameter *R*^2^ (0.217–0.717), i.e., the poor agreement between experimental and model values. They hypothesized that the thickness of the boundary layer between the particle and the water phase can affect the diffusion of the insecticides to/into the particle, the so-called thermal diffusion. However, since they found a poor effect in the application of WMM, they assumed that Fick diffusion in the aqueous phase could be accompanied by other attenuations of the overall mass transfer rate, such as van der Waals forces between the insecticide molecule and the soil particle surface [33]. In our research, much higher values of *I*^sor^ (62.05–94.13 mg/kg) were obtained, compared to Aseperi et al. [33] (4.32–10.18 mg/kg), indicating a greater thickness of the boundary layer, and, thus, a stronger influence of intraparticle diffusion on the sorption processes. Furthermore, the same study showed that the thickness of the boundary layer depended on the amount of soil OM, which was also proven by our study. On the contrary, Fernandez-Bayo et al., who studied the sorption of imidacloprid in Spanish soils, found that the boundary layer surrounding the soil particles had an important effect on the initial kinetics of imidacloprid sorption [44]; that is, the sorption processes in these soils could be well described by the WMM model. The values of the parameters *I*^sor^ and *k*^sor^ obtained in their study were in the range of 3.35 to 3.68 mg/kg and from 0.026 to 0.037, respectively. Rodriguez-Liebana et al. analysed the sorption kinetics of thiacloprid in Mediterranean soils, and they successfully applied the WMM model for the description of sorption behaviour with very high *R*^2^ (0.97) [48]. They also reported lower *I*^sor^ and *k*^sor^ parameter values (2.85 and 0.039) compared to our study. According to the acetamiprid and thiacloprid sorption results obtained in this study, the *C*^sor^ parameters were of positive values, indicating the rapid sorption process in a short period of time, and therefore showing that the sorption is not only controlled by intraparticle diffusion, but also by boundary diffusion [48].

#### 2.2.4. Two-Site Model (TSM)

The two-site model provided an excellent fit to the experimental data, as indicated by the high *R*^2^ values (>0.9999) and the lowest errors (SRMSE = 0.0011–0.0131; err-% = 0.10–1.17%) compared to all other tested models (Table 2, Table 3 and Table 4). Furthermore, as previously mentioned, the sorption kinetics of both insecticides take place as two-phase processes, where the rate of each phase differs significantly. This means that, if the initial phase is rapid and instantaneous, it takes place in the first few hours of the reaction, followed by a slow phase. An inverse process is also possible, where an initial, longer-lasting sorption process is observed. It can thus be inferred that this biphasic model should be used for the description of acetamiprid and thiacloprid sorption in all soils. The kinetics parameters for acetamiprid and thiacloprid obtained by TSM are presented in Table 4.

With this model, four parameters were optimized: reaction rate constants (*k*_1_^sor^ and *k*_2_^sor^) and amount of sorbed insecticides (*q*_1_^sor^ and *q*_2_^sor^) for each site type, respectively. Very similar behaviour in the acetamiprid sorption process was observed in soils S1, S3 and S4, where the sorption rates were evidently higher in the second compartment sites (Table 4). The sorption rate was significantly higher in the first compartment sites only in the S2 soil. Although the sorption of thiacloprid was similar to that of acetamiprid, several differences were observed. In soils S1 and S2, the thiacloprid sorption rates for both site types were significantly higher, while in soil S3 a higher thiacloprid sorption rate was achieved only for the first compartment site. Furthermore, it is interesting that in soil S4, sorption of acetamiprid occurred at higher rates than sorption of thiacloprid. According to the previously mentioned facts, the sorbed amounts of acetamiprid in the second compartment were in the order S1 (66.44 mg/kg) > S4 (56.63 mg/kg) > S3 (56.32 mg/kg) > S2 (6.65 mg/kg). The same increasing trend for the amount of thiacloprid in the second compartment was also achieved in the analysed soils, with the difference being that sorbed amounts of thiacloprid were on average 1.7 fold higher compared to acetamiprid.

Regarding the desorption process, the TSM again appears to give the best description for both insecticides in all analysed soils, compared to the remaining tested models [high *R*^2^ (0.9999), low SRMSE (0.0007–0.0077) and err-% (0.06–0.73%), Table 2, Table 3 and Table 4]. The application of TSM as a superior model in the explaining of the applied insecticides desorption processes indicated that desorption also takes place in two compartments, characterized by different reaction rates. The desorption kinetics parameters estimated by TSM for both insecticides are also presented in Table 4. Contrary to the acetamiprid sorption processes, the desorption rate (*k*_1_^des^) was more intense in the 1st compartment in soils S1, S3, and S4. Comparing desorption rate values, *k*_1_^des^ and *k*_2_^des^, the rate of difference was highest in soil S3 (69 fold), slightly lower in soil S3 (65 fold) and the lowest in soil S1 (13 fold). Only in soil S2 was the *k*_2_^des^ of acetamiprid higher in the 2nd compartment, by 21 fold. Thiacloprid desorption behaviour was almost same as in the case of acetamiprid, but only in soils S1 and S2. In soil S1, *k*_1_^des^ was 31 times faster than in the second compartment, while in soils S2, S3, and S4, *k*_2_^des^ was 31, 33 and 15 times faster than in the first compartment. The highest amount of acetamiprid desorbed in the first compartment (*q*_1_^des^) was observed in soil S4, and the lowest in soil S1, while in the second compartment the highest *q*_2_^des^ of acetamiprid was observed in soil S2, and the lowest in soil S1. Contrarily to acetamiprid, the values for the amount of thiacloprid desorbed in the first compartment (*q*_1_^des^) in all soils was higher than those obtained in the second one (*q*_1_^des^ > *q*_2_^des^).

The values of all statistical indices for the analysed models are presented in Table 2 and Table 3.

### 2.3. Effect of Physico-Chemical Soil Characteristics on Acetamiprid/Thiacloprid Sorption/Desorption Parameters

Analyses of the effects of the physico-chemical soil properties on the sorption/desorption parameters estimated by the TSM are presented in Table 5 and Table 6. The correlation of acetamiprid and thiacloprid *k*_1_^sor^ with TOC, ratio 465/665, and ratio C/H was negative but not statistically significant. Likewise, acetamiprid *k*_2_^sor^ positively correlated with TOC, ratio 465/665, and ratio C/H, however none of the correlations were statistically significant. However, thiacloprid *k*_2_^sor^ showed a strong positive and statistically significant dependence on the TOC (0.956; *p* = 0.044) and ratio 465/665 (0.983; *p* = 0.017). A strong correlation of *k*_2_^sor^ with HA and CEC was also observed, but it was not statistically significant. Regarding the insecticide amounts in each compartment, a strong positive impact of TOC (0.966; *p* = 0.034) and ratio C/H (0.993; *p* = 0.001) on thiacloprid *q*_2_^sor^ was found. Thiacloprid *k*_1_^des^ was significantly positively correlated with HA (0.990; *p* = 0.010), CEC (0.997; *p* = 0.003) and ratio C/N (0.996; *p* = 0.004), as was *k*_2_^des^ with clay content (0.976; *p* = 0.024), Table 5.), while significant negative correlations were found between *k*_2_^des^ and ratio C/N (−0.965; *p* = 0.035), and *q*_2_^des^ and fulvic acids content (−0.984; *p* = 0.016). Correlations of acetamiprid *k*_1_^des^ with TOC, humic acids, and ratio 465/665 were positive, with a strong but statistically insignificant impact on the ratio 465/665 (0.821; *p* > 0.05). The same physico-chemical soil parameters had a negative impact on the *k*_2_^des^.

Salvestrini et al. [51] applied the TSM in the analysis of the imidacloprid sorption kinetics in Northern Italian soils, where the model was able to describe the complete experimental data well (*R*^2^ = 0.81–0.97). They hypothesized that the imidacloprid sorption process consists of a rapid phase of molecules binding to soil colloids (<1 h), followed by a much slower phase (several weeks). They gave a possible explanation for such imidacloprid sorption behaviour, i.e., that dissolved imidacloprid molecules in a bulk solution were rapidly and reversibly bound to the external soil colloid surface, and that they then could diffuse and bind to the interior of the pores. That is, in the rapid phase, the reversible imidacloprid binding prevailed over diffusion-controlled processes; this is also confirmed by the desorption process completing within a few hours after the start of sorption, while later it was less effective.

### 2.4. Principal Component Analysis

**Figure 3:**
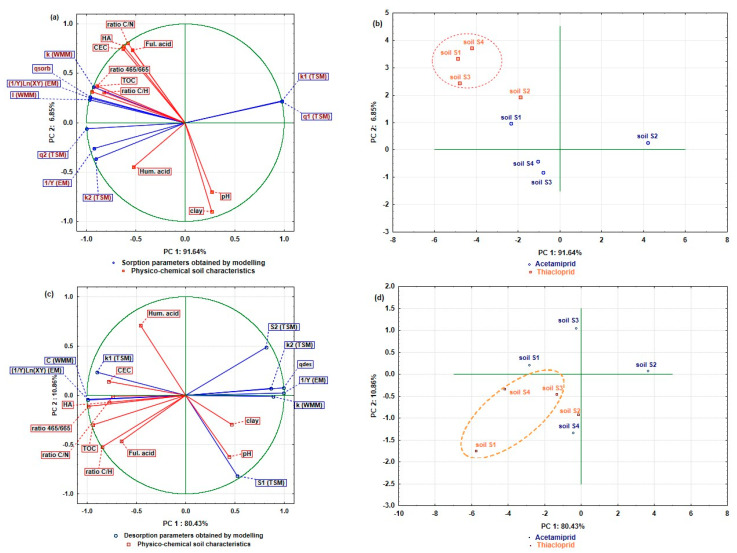
Effect of physico-chemical soil characteristics on acetamiprid/thiacloprid sorption (**a**,**b**) and desorption (**c**,**d**) parameters obtained by mathematical modelling represented by principal component analysis (PCA) (N = 1200; pooled data; 4 soils × 3 replication × 10 soil characteristics × 10 sorption parameters) in soils S1 to S4 represented by two main components (PC1 and PC2) (**a**,**c**) projections of the variables: active (sorption parameters) and supplemental (soil characteristics); (**b**,**d**) Projections on cases (soils) on the factor-plane.

Effect of physico-chemical soil characteristics on acetamiprid/thiacloprid sorption/desorption parameters obtained by mathematical modelling were analysed by principal component analysis (PCA) (N = 1080; pooled data; 4 soils × 3 replication × 10 soil characteristics × 9 sorption parameters for each process). The results are depicted in Figure 3a–d, for sorption and desorption processes, respectively.

Evaluated insecticide sorption and desorption parameters were used as variables for the analysis, soil characteristics as supplementary variables, insecticides as the active case variable and soils as a group variable. According to the applied Kasier–Gutman method and Cattell scree test, two main components (PC1 and PC2) were used for the PC model analysis. With PC1 and PC2, it is possible to explain 98.49% of the total variance for the insecticides’ sorption processes, while for the desorption, this percentage is slightly lower and amounts to 91.29%. As can be seen from Figure 3a, PC1 explains almost the entire model variance, to the amount of 91.64%, while the remaining part of 6.85% is made up by PC2. Figure 3b shows the projection of the analysed soils, depending on the estimated sorption parameters and applied insecticides.

Interpretation of main components (PC1 and PC2) was performed using eigenvectors. Sorption PC1 was defined with *k*_1(TSM)_ (0.97), *q*_1(TSM)_ (0.98), *q*_2(TSM)_ (−0.99), (1/Y)Ln(XY)_(EM)_ (−0.97), *I*_(WMM)_(−0.97), while *k*_2(TSM)_ (−0.91), *k*_(WMM)_ (−0.93) and 1/Y_(EM)_ (−0.92) have defined PC2. Of the physico-chemical soil characteristics, as supplementary variables, TOC (−0.90), ratio 465/665 (−0.95) and ratio C/H (−0.83) had negative effects on PC1, while the effect of Hum. acids was negative and less pronounced (−0.52). On the PC2 definition, C/N (0.81), HA (0.77) and CEC (0.75) showed positive and medium strong effects, while the weakest influence was that of Ful. acids (0.73). Contrarily, the influence of clay on the PC2 was strong and negative (−0.90), while pH had a moderately strong and negative effect (−0.70). Comparing the variable distribution of the sorption parameters and physico-chemical characteristics of the soil (Figure 3a), with the soils depending on the acetamiprid and thiacloprid sorption processes (Figure 3b), it can be observed that the differences in the sorption mechanism of both insecticides on analysed soils are notable, and that each insecticide sorbs differently to soil colloids depending on the physico-chemical characteristics of the soil, which can significantly affect the mechanisms of the process. On the factor plane (Figure 3a,b), the second quadrant contains the sorption parameters *k*_1(TSM)_ and *q*_1(TSM)_ and the soil S2 on which the acetamiprid sorption takes place. This result leads to the inference that the sorption rate and the sorbed amount in the first compartment play a dominant role in the overall acetamiprid sorption process on the soil S2. Furthermore, in the definition of the positive (right) side of PC1, the supplementary variables—namely clay and pH—dominate; this is also where the soil S2 is presented, on which acetamiprid sorption takes place, indicating that the mentioned soil characteristics are dominant in the regulation of the sorption process in this soil. In the fourth quadrant are the parameters *k*_2(TSM)_ and *q*_2(TSM)_, and soils S3 and S4 with sorbed acetamiprid. In these soils, acetamiprid sorption is a biphasic process, in which the sorption rate has a dominant role in the second compartment. Parameter 1/Y_(EM)_ is located in the same quadrant, indicating that the coverage of acetamiprid molecules was intense and directly affected the sorption or chemisorption processes, as well as the activation energy of the process itself. Likewise, the higher acetamiprid sorption capacity is influenced by the larger amount of Hum. acids. The largest number of estimated sorption parameters (*q*^sor^, (1/Y)Ln(XY)_(EM)_, *k*_(WMM)_ and *I*_(WMM)_) and physico-chemical soil characteristics (TOC, fulvic acids, ratio C/N, ratio 465/665, ratio C/H, HA, CEC) are in the third quadrant of the factor plane (Figure 3a), as well as soil S1 with sorbed acetamiprid, and all analysed soils with sorbed thiacloprid (Figure 3b). Considering the *q*^sor^ parameter, it can be observed that the thiacloprid sorption was more intense compared to acetamiprid, with sorption capacity decreasing in the order soil S1 > S3 > S4. Parameter 1/Y_(EM)_ indicates that the overall rate of thiacloprid sorption is primarily determined by the fast reaction phase, as well as the acetamiprid sorption in the soil S1. The parameters *k*_(WMM)_ and *I*_(WMM)_, located in the third quadrant, indicate the thickness of the boundary layer and the influence of intraparticle diffusion on the sorption process. The influence of the mentioned parameters was more pronounced in soils S1, S3 and S4 with thiacloprid sorption, and in soil S1 where acetamiprid sorption took place. In these soils, sorption processes were regulated by the TOC, the ratio 465/665, and the C/H ratio. Furthermore, HA and CEC regulated the sorption processes, although they showed a somewhat weaker effect.

Desorption processes are presented on the factorial plane of Figure 3c,d. With the PC1 and PC2 retained in the model, it is possible to explain 80.43% and 10.86% of the total variance, respectively. All estimated desorption parameters, except *q*_1(TSM)_, define the PC1. A strong positive effect on PC1 is observed by *q*^des^ (0.99) and 1/Y_(EM)_ (0.99), while the parameters *k*_(WMM)_ (0.89), *k*_2(TSM)_ (0.87), and *q*_2(TSM)_ (0.82) were positive but medium strength. On the contrary, parameters *I*_(WMM)_ (−0.99), (1/Y)Ln(XY)_(EM)_ (−0.99), and *k*_1(TSM)_ (−0.90) showed a strong negative influence on the definition of PC1. Parameter *q*_1(TSM)_ (−0.82) clustered around PC2 with negative and moderate influence. In contrast to the sorption processes, where variables of the soil physico-chemical characteristics were uniformly distributed around PC1 and PC2, in the desorption processes, most variables (8) were defined by PC1. The majority of effects were negative and strong (ratio 465/665 (−0.99) and TOC (−0.94)), then medium (ratio C/H (−0.85), CEC (−0.78), HA (−0.77), ratio C/N (−0.74) and fulvic acids (−0.65)). The clay content (0.47) was variable, with a positive weak effect on the definition of PC1. Variables, including pH with a negative effect (−0.62), and Hum. acids with a positive effect (0.71), are grouped around PC2. Examining Figure 3c,d, the parameters *q*_1(TSM)_, *q*_2(TSM)_ and *k*_2(TSM)_ are situated on the right side of PC1, along with the soil S2 containing acetamiprid desorption, indicating, on the biphasic process, where the desorption rate in the second compartment had had a distinct influence on the overall rate of the process. Likewise, on the same side of PC1 are also located the desorption parameters 1/Y_(EM)_ and *k*_(WMM)_, leading to the assumption that, in soil S2, the acetamiprid molecules weakly bound to soil colloids were more susceptible to desorption, compared to the remaining analysed soils. This assumption is confirmed by the *q*^des^ parameter, located in the second quadrant, as well as the soil S2, proving that the acetamiprid desorption was the most pronounced in this soil. The pH and clay content showed a dominant effect on the desorption processes in soil S2. In the third quadrant, parameter *k*_1(TSM)_, and the soils S1 and S3, where the desorption of acetamiprid occurred, are located. The above indicates that a biphasic desorption process takes place in these soils as well, where the desorption rate in the first compartment had the strongest influence on the overall rate of the process. Furthermore, in soil S3, desorption is dominantly dependent on the level of Hum. acids, while in soil S1, the process is regulated by the amount of CEC. Moreover, a stronger effect of the first phase rate on the overall desorption rate was observed in soil S1, compared to soil S3. Two desorption parameters were estimated, (1/Y)Ln(XY)_(EM)_ and *I*_(WMM)_), along with six supplementary variables (TOC, fulvic acids, ratio C/N, ratio 465/665, ratio C/H, and HA), as well as soils S1–S4 and soil S4, where thiacloprid and acetamiprid desorption occurred. Such a distribution of variables indicates that, in the mentioned soils, especially in soil S4 where thiacloprid desorption took place, the processes depend on the thickness of the boundary layer and intraparticle diffusion. The strongest impact on the thiacloprid desorption processes in soil S4 was achieved by HA, ratio 465/665 and ratio C/N. Furthermore, in soils S1 and S3, the intensity of thiacloprid desorption was dominantly influenced by TOC, fulvic acid levels and C/H ratio.

## 3. Discussion

The two insecticides analysed in this study have very similar sizes and chemical structures, as shown in Table 7. Nonetheless, some of their basic physical properties—such as water solubility and lipophilicity (described by *K*ow)—differ greatly. The water solubility of thiacloprid is an order of magnitude lower than the solubility of acetamiprid, and it has the lowest value of all neonics [42]. Its solubility is not influenced by pH in the range between 4 and 9, and it has no acidic or basic properties in an aqueous solution [52]. Additionally, thiacloprid is three times more lipophilic than acetamiprid. Such behaviour is in gross disproportion with the fact that thiacloprid is able to create more hydrogen bonds than acetamiprid (3 vs. 4, Table 7) [53]. Note that the molecules are of similar sizes, so the size differences are excluded as a factor. Both compounds contain a 6-chloropyridin aromatic unit on one side, and a nitrile group on the opposite side of the molecule. Negative charges are located on both sides, as designated by orange/red shadings [47]. The location of the positive charge differs due to a key structural difference, that is, the presence of a thiazolidine ring in thiacloprid. It is known that thiazolidine moiety is very weakly soluble in water if it does not carry aromatic or higher aliphatic groups on the nitrogen atom [54]. The fact that, in thiacloprid, the thiazolidine nitrogen does carry a large aromatic unit could thus contribute to its poor water solubility. The presence of the ring structure (as opposed to non-cyclic moieties) has been recognized as a major factor contributing to the increased lipophilicity [4,55,56]. Additionally, the thiazolidine ring contains a sulphur atom, which was also found to have the same effect [4,55,56].

Additionally, the data from the literature suggest that the acid–base properties of the two insecticides differ, with acetamiprid having s p*K*_a_ value of 0.7, while thiacloprid was reported not to dissociate [17,30,31,42]. This extremely low p*K*_a_ implies that acetamiprid is a strong acid (and is listed as such [17]), and, consequently, that at a typical soil pH, the acetamiprid exists in an ionic form, which would present a major contribution to the differences in sorption/kinetic behaviour. However, connecting acetamiprid’s chemical structure to strong acidic properties is not obvious nor logical; there are no functional groups that would dissociate at such low pH. In fact, the presence of nitrogen atoms make both compounds weak bases. Thus, this p*K*_a_ value designates the acidity of the molecule’s protonated form (protomer). Protonation at a particular site in a molecule depends on a particular atom’s proton affinity, but in most cases, the proton affinity of different sites in a molecule is the result of a synergistic effect of all the functional groups present [57]. Here, both compounds contain four nitrogen atoms in similar surroundings: one *sp* hybridized (the nitrile group N), two *sp*^2^ hybridized (one of which is a pyridine N), and a single *sp*^3^ hybridized N. The most pronounced difference can be noticed for *sp*^3^ N, since it is located within the ring structure and in the vicinity of sulphur in thiacloprid, but not in acetamiprid. Hence, presumably, this *sp*^3^ N is the one being protonated at low pH. Nevertheless, while the protonation of nitro-containing neonicotinoids seems to be well researched [57,58], to the best of our knowledge, equivalent studies on acetamiprid could not be found to corroborate these assumptions.

The described differences in the molecular structure, resulting in thiacloprid’s poor water solubility, higher lipophilicity, and different acid–base properties, provide an explanation of the differences in sorption/desorption behaviour of the two compounds. Our results showed a more rapid sorption of thiacloprid compared to acetamiprid, and a higher amount of thiacloprid sorbed at equilibrium, for all soils. Having less inclination to be surrounded by water molecules, and a lipophilicity three times greater, seems like a plausible explanation for such behaviour. For thiacloprid, the equilibrium is reached suddenly, indicating that thiacloprid sorbed on easily accessible external sites, effectively without any intra-particle diffusion. Since the amount of thiacloprid sorbed at equilibrium is proportional to the amount of organic matter, it is likely that those sites are predominantly located there. On the other hand, after the initial rapid sorption phase, acetamiprid reached equilibrium gradually, indicating that intra-particle diffusion is a relevant process in the case of this insecticide. Despite longer equilibrium times, the proportion sorbed at equilibrium never exceeded that of thiacloprid. Desorption results showed that acetamiprid is more easily and more thoroughly desorbed than thiacloprid in all soils (the average retention for thiacloprid was 94%, versus 84% for acetamiprid), which is also in accord with its higher water solubility. Additionally, the results showed that only small amounts of the insecticides were desorbed, which can be attributed to the fact that both thiacloprid and acetamiprid readily degrade in soil [42].

## 4. Materials and Methods

### 4.1. Chemicals

Insecticide analytical standards (at purity ≥ 99.0%) of acetamiprid and thiacloprid (Dr. Ehrenstorfer GmbH, Augsburg, Germany) were used in this study. Stock insecticide solutions (1 mg/mL) were prepared by dissolving the required amount of each insecticide in HPLC grade acetonitrile (J.T. Baker, Deventer, Holland), while for the sorption/desorption kinetics experiments, stock standard solutions were diluted with 0.01 M aqueous calcium chloride (CaCl_2_) solution to obtain the insecticide concentration of 30 mg/L. Sodium acetate (CH_3_COONa), CaCl_2_, sodium hydroxide (NaOH), sodium pyrophosphate (Na_4_P_2_O_7_ × 10 H_2_0), potassium dichromate (K_2_Cr_2_O_7_), sulfuric acid (H_2_SO_4_), and ammonium acetate (CH_3_COONH_4_) were purchased from Kemika (Zagreb, Croatia), while hydrochloric acid (HCl) and glucose (C_6_H_12_O_6_) were from Merck (Darmstadt, Germany). A standard pesticide analysis kit (Pestizidruck kit RESTEK 31971 LC Multi) was purchased from Restek (Bellefonte, PA, USA), methanol (CH_3_OH) hypergrade for LC/MS from Supelco (Darmstad, Germany), ammonium format (HCOONH_4_) from Fluka (Deisenhofen, Germany), and the certified EDTA standard containing 41.06 wt% of C, 5.51 wt% of H, and 9.56 wt% of N from LECO Corporation (Saint Joseph, MI, USA). Deionized water was prepared using an Ultrapure Water Systems (Ultra Clear™ TP ED TWF, Evoqua Water Technologies (Guenzburg, Germany). The chemical structure of insecticides used with their physico-chemical properties are presented in Table 7.

### 4.2. Experimental Soils and Physico-Chemical Characteristics Soil Determination

**Figure 4:**
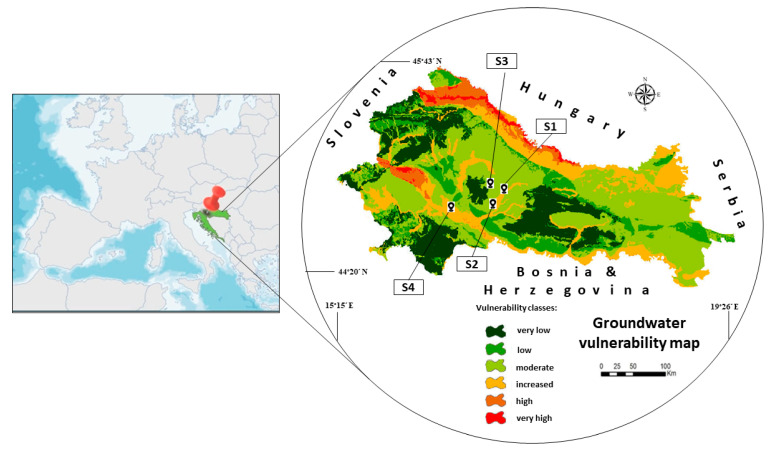
Graphic location of the soil sampling sites (S1–S4) and their position on the groundwater vulnerability map.

Soil samples were collected from the localities of two Croatian counties: Požega-Slavonska (area around cities of Pakrac and Lipik) and Sisak-Moslavina (area around the city of Kutina) (Figure 4), where neonicotinoids are often used to protect sugar beet plantations from various pests. Sampling was carried out according to the Standard Sampling Procedure ([59]), respecting the principles of sampling: randomness, independence, impartiality, and representativeness. One forest soil, Sample S1 (city Pakrac; GPS coordinates: 45°49′ N, 17°08′ E), one lake sediment, Sample S2 (city Lipik, lake Raminac; GPS coordinates: 45°42′ N, 17°13′ E), and two agricultural soils, Sample S3 (Ploština; GPS coordinates: 45°29′ N, 17°07′ E) and Sample S4 (city Kutina, GPS coordinates 45°47′ N, 16°80′ E) are used in the study. Soils were collected on 0.5 ha of the soil surface using the “diagonal” method [59]. The sampling procedure was carried out with a stainless-steel probe from the A horizon at a depth of up to 30 cm in such a way that one sample was taken at each top of the rectangle and one sample at the intersection of the diagonals of the rectangle. The lake sediment sample was taken from the 100 m long coastal strip of the lake. Thus, five individual samples of 2 kg were taken from each locality in plastic boxes with lids. The soils were air-dried in the laboratory, crushed, and sieved through a sieve (Ø 2 mm). The soils from each location were mixed, homogenized, and “quartered” to obtain a representative sample, and then stored at a temperature of 20 ± 1 °C prior to analysis. Prior to conducting sorption/desorption kinetic processes, the soils were checked for acetamiprid and thiacloprid residue by HPLC-MS/MS.

The soil solid phase texture (fragmentation) was determined by sodium pyrophosphate [60]. Actual and substitutional acidity were analysed in soil and water suspension as well as in soil and 0.01 M CaCl_2_ suspension (1:2.5 *w*/*V*), while hydrolytic acidity (HA) was determined by basic salt (CH_3_COONa), and by alkali titration of the soil suspension [61]. Cation exchange capacity (CEC) was determined by the replacement of Ca^2+^, Mg^2+^, K^+^ and Na^+^ ions with NH_4_^+^, whose amounts were analysed on an AA Spectrophotometer (Perkin Elmer Analyst, Waltham, MA, USA) ([62] (Sumner and Miller, 1996). The humus content was determined spectrophotometrically (Spectroquant^®^Pharo 100, Merck, Darmstadt, Germany) after oxidation with K_2_Cr_2_O_7_ and concentrated H_2_SO_4_) ([63]), while the total organic carbon content (TOC) was analysed according to the HRN EN 15936 (2013) method by burning at 900 °C using a TOC module equipped with a Non-Dispersive InfraRed detector (Shimatzu TOC module, Kyoto, Japan). During the burning process in a stream of oxygen, CO_2_ is released, the amount of which is directly proportional to the amount of C in the soil sample. Prior to burning, to remove inorganic carbon, the soil sample was treated with non-oxidizing mineral acid (HNO_3_). The amount of TOC is expressed as the amount of C (%) in dry matter of the soil. Contents of humic (C_HA_) and fulvic acids (C_FA_) were determined according to the method proposed by Kononova and Belcikova ([64]), after alkaline extraction. The composition, i.e., aliphaticity or aromaticity of the extracted humic and fulvic acids was determined spectrophotometrically at 465 and 665 nm. Determination of total C, H and N contents was performed according to the method HR EN 15407 (2011) by CHN analyser (Leco 628 CHNS, (Saint Joseph, MI, USA) equipped with InfraRed (IR) detector for C and H analysis and Thermal Conductivity (TC) detector for N analysis. The C, H and N content in soil samples was expressed as a percentage (%) of each element on the dry matter of the soil. As a standard compound for calibration curve preparation, EDTA was used. Each measurement of the standard or the sample was made in triplicate. Physico-chemical properties of the experimental soils are depicted in Table 1.

### 4.3. Sorption/Desorption Kinetic Experiments

Acetamiprid and thiacloprid sorption by soils was quantified using the standard batch equilibrium method summarized in OECD Technical Guideline 106 [65]. From the insecticide stock solution (1 mg/mL), the working solutions (30 mg/L) of each insecticide were prepared by diluting with 0.01 M CaCl_2_, which was used as a background electrolyte to maintain a constant ionic strength and to promote flocculation. Experiments were performed in triplicate, with samples of 5,0 g of air-dried soils, mixed with 25 mL aliquot of 30 mg/L aqueous solution of each insecticide in 50 mL centrifuge tubes. The tubes were shaken on a horizontal shaker (Heidolph promax 2020, Schwabach, Germany) at 120 rpm, after which the 0.25 mL of suspension was removed, after 0.25, 0.5, 1, 3, 6, 12, 24, 48, and 96 h, for insecticide residue analysis. The removed suspensions were centrifuged at 4000 rpm for 3 min (Universal 320 R Hettich, Tuttlingen, Germany) and each supernatant was filtrated through a 0.22 µm membrane pore-size Millipore filter (Merck, Darmstadt, Germany). After filtration, the aqueous phase was analysed by HPLC-MS/MS. Blank samples of acetamiprid and thiacloprid were prepared and treated in the same way as the samples, one without soil and one without pesticide, to avoid possible sorption on the filters or centrifuge tubes, degradation, and volatilization during the experiment. Control samples were used for each series of experiment.

The amount of each insecticide sorbed at any soil-solution contact time ($\Delta t_{n}=t_{n}$−$t_{n-1}$) for kinetic sorption experiments was calculated by Equation (1).

(1) $$m_{s}^{sor}\left(\Delta t_{n}\right)=m_{m}^{sor}\left(t_{n-1}\right)\cdot \left(\frac{V_{0}-\left(n-2\right)\cdot v_{a}^{A}}{v_{a}^{A}}\right)-m_{m}^{sor}\left(t_{n}\right)\cdot \left(\frac{V_{0}-\left(n-1\right)\cdot v_{a}^{A}}{v_{a}^{A}}\right)$$

where $m_{s}^{sor}\left(\Delta t_{n}\right)$ and $m_{m}^{sor}\left(t_{n}\right)$ were the masses of insecticides sorbed during the time intervals ($\Delta t_{n})$ or measured in an aliquot ${(v}_{a}^{A})$ at the time points $\left(t_{n}\right)$, while *V*_0_ is initial volume of the insecticide solution. After the sorption process, the desorption process was carried out in such a way that the volume of solution was removed after 96 h of sorption processes and replaced with 25 mL of 0.01 M CaCl_2_ solution. The soil-solution mixture was shaken again for 0.25, 0.5, 1, 3, 6, 12, 24, 48 and 96 h, centrifuged, and 0.25 mL of supernatant was removed for analysis. The rest of the procedure was the same as for the sorption process.

The amount of insecticide desorbed by the soil during each time interval ($\Delta t_{n}=t_{n}$− $t_{n-1}$) was calculated by Equations (2) and (3).

(2) $$m_{aq}^{des}\left(\Delta t_{n}\right)=\left[m_{m}^{des}\left(t_{n}\right)\cdot \left(\frac{V_{T}}{v_{a}^{D}}\right)-m_{aq}^{A}\left(\frac{V_{T}-\left(n-1\right)\cdot v_{a}^{D}}{V_{T}}\right)-\sum_{i=1,n\neq 1}^{n-1}\left(\frac{{(V}_{T}-\left(n-i\right)\cdot v_{a}^{D})}{V_{T}}\cdot m_{aq}^{des}\left({\Delta t}_{i}\right)\right)\right]$$

(3) $$m_{s}^{des}\left(t_{n}\right)=m_{s}^{sor}\left(eq\right)-\sum_{i=1,n\neq 1}^{n}m_{aq}^{des}\left({\Delta t}_{i}\right)$$

In Equation (2) and (3) $m_{s}^{des}\left(\Delta t_{n}\right)$, $m_{aq}^{des}\left(\Delta t_{n}\right)$ and $m_{m}^{des}\left(t_{n}\right)$ represent the masses of insecticides remaining sorbed on the soil after the time interval $\Delta t_{n}$, desorbed during the time interval $\Delta t_{n}$ and measured in an aliquot ($v_{a}^{D}$) at time point $t_{n}$, $V_{T}$ is the total volume of the aqueous phase, and $m_{aq}^{A}$ is mass of the insecticide left over from the sorption equilibrium.

### 4.4. Instrumentation and Operating Conditions

Prior to their determination on an AAS800 (Perkin Elmer Analyst, Waltham, MA, USA) equipped with Autosampler AS 800 Perkin Elmer and Software AA WinLab32, the cations (Na^+^, K^+^, Mg^2+^, Ca^2+^) were extracted from the soil by digestion (conc. HNO_3_) and combustion in a microwave oven (MLS-1200 Mega Microwave Digestion System, Milestone, Sorisole, Italy). The experimental conditions in the oven were set as follows: 5 min—300 W, 0.5 min—0 W, 5 min—600 W, 1 min—ventilation. After cooling, the homogenous suspension was diluted with ultrapure water and analysed on AAS. Ca^2+^, Mg^2+^, K^+^, and Na^+^ were determined at 422.7, 285.2, 766.5 and 589.0 nm, respectively and quantified by external standard method. The calibration curves (five concentrations in triplicate) were linear in the range 1–10 mg/L for Ca^2+^, 0.5–2 mg/L for Mg^2+^, 0.5–3 mg/L for Na^+^ with a regression coefficient of *R*^2^ > 0.9950. Limit of detection (LOD) and limit of quantification (LOQ) were calculated by expressions: LOD = 3.3 *r*/*S* and LOQ = 10 *r*/*S*, where *r* and *S* represent the standard deviation of the blank (calculated over ten injections of the blank), and *S* is the slope of the calibration curve. The quantification limits were: 10 mg/kg for Ca, 5 mg/kg for Mg and Na. All analysis included blank, standard solutions, samples were performed in triplicate, and results were expressed in mg/kg on the dry mass of soil.

The acetamiprid and thiacloprid residues were determined by HPLC-MS/MS (Exion LC, Concord, Ontario, Canada) using an Phenomenex Kinetex C18 analytical column (100 × 2.1 mm i.d., 2.6 µm particle size, 100 Å pore size, Phenomenex, Torrance, CA, USA). Chromatographic analyses were performed using mobile phase of A solution: 90% H_2_O, 10% CH_3_OH + 5 mM HCOONH_4_ and mobile phase of B solution: 10% H_2_O, 90% CH_3_OH + 5 mM HCOONH_4_. After stabilization (30 min at 40 °C), when mobile phases were passed through the column at a volume ratio of 50:50, insecticide analysis was performed by gradient elution. The total run time was 20 min, the flow rate was set at 0.4 mL/min, and the injection volume was 30 μL. The gradient elution program was as follows: 0–1 min, 98% mobile phase A and 2% mobile phase B; 15–18 min, 2% mobile phase A and 98% mobile phase B; 18.05–20.00, 98% mobile phase A and 2% mobile phase B. Under these conditions, the acetamiprid and thiacloprid retention times were 6.05 and 6.90 min, respectively.

### 4.5. MS/MS (Detector AB SCIEX 4500 QTRAP) Conditions

The detection of the analysed insecticides was carried out with a quadrupole mass spectrometer with electrospray ionisation (ESI), operating in a positive ion mode. The MS conditions were as follows: the ionization voltage was optimized at 5500 V, the ion source temperature was 400 °C, and the pressures of the ion source spray gases 1 and 2 were 50 and 55 psi, respectively. An increased confidence in the analytical results was provided by the mass spectral library for the identification of compounds (Enhanced Product Ion (EPI) mode (AB Sciex, Framingham, MA, USA)), which significantly reduced the risk of false positive results. Data collection in multiple reaction monitoring (MRM) mode was optimized after direct infusion of each individual standard solution into the detector. Therefore, two ion transitions were selected for each compound, quantifier and qualifier MRM. Two fragments of the analysed insecticides were monitored, from which the fragment with more intense peak was used for quantification, while the second peak was used for confirmation. Ion transitions for both insecticides, along with declustering potential (DP), collision energy (CE), exit potential (EP), and collision cell entrance potential (CEP), are listed in Table 8. The identification and data processing of pesticide residues were made through the Analyst^®^ 1.6.1 Software (AB Sciex, Framingham, MA, USA). For linearity, the calibration curves of acetamiprid and thiacloprid were prepared at six different concentration levels (from 1 to 100 ng/mL) in triplicate. The linear regression and squared correlation coefficient (*R*^2^) of the calibration curves were *R*^2^ > 0.9999. A calibration curve was made before each sequence of samples. In each sequence of samples, analysis was performed at the beginning, in the middle and at the end. An insecticide standard was used in order to observe if there was a possible deviation in the intensity of the analytes. If the accuracy was over 20%, then a correction was carried out for those samples in the sequence. Since there was no sample preparation (extraction), the analytes in sample were analysed directly, and the accuracy of the previously prepared pesticide solutions was checked (6 samples in concentration of 50 ng/mL), ranging from 94 to 106% for acetamiprid (RSD = 5%) and from 97 to 104% for thiacloprid (RSD = 3.2%). The LOQ was 0.1 ng/mL or below for both insecticides, which allows the dilution of sample extracts and the reduction of matrix effects. The LOD for acetamiprid was 0.03 ng/mL, while for thiacloprid was 0.024 ng/mL. To keep the concentrations of the tested insecticides in the validated measuring range, the samples were diluted (dilution factors 10 and 50 to 1000 μL of final volume).

### 4.6. Sorption/Desorption Kinetic Models

Models used for description of acetamiprid and thiacloprid sorption/desorption kinetic processes in soils were: 

#### 4.6.1. Lagergren’s Pseudo First-Order Model (PFOM)

This model assumes that the insecticides sorption/desorption processes on soil colloids take place by the mechanism of first-order kinetics, what is shown by the differential Equation (4):

(4) $$\frac{dq_{t}^{sor}}{dt}=k_{1}\left(q_{eq}^{sor}-q_{t}^{sor}\right)$$

where $q_{eq}^{sor}$ and $q_{t}^{sor}$ (mg/kg) are the insecticide sorption capacities at equilibrium and amount sorbed at time *t* (h), respectively, while *k*_1_ is the first order reaction rate constant of (1/h). Integrating Equation (4) to the boundary conditions *q*_t_^sor^ = 0 at *t* = 0 and *q*_t_^sor^ = *q*_t_^sor^ at *t* = *t* expression becomes:

(5) $$q_{t}^{sor}=q_{eq}^{sor}\left(1-e^{-k_{1}t}\right)$$

Desorption insecticides process in soil according to PFOM is shown using Equation (6):

(6) $$-\frac{dq_{t}^{des}}{dt}=k_{1}\left(q_{t}^{des}-q_{eq}^{des}\right)$$

where $q_{eq}^{des}$ is amount of insecticide sorbed at desorption equilibrium (mg/kg), $q_{t}^{des}$ is the amount of insecticide sorbed on the surface of soil (mg/kg) at any time *t*.

Integrating Equation (6) for the boundary conditions $q_{t}^{des}$=$q_{eq}^{sor}$ and *t* = 0 Equation (7) was obtained:

(7) $$q_{t}^{des}={q_{eq}^{des}+\left(q_{eq}^{sor}{-q}_{eq}^{des}\right)e}^{-k_{1}t}$$

#### 4.6.2. Nonequlibrium Two-Site Model (TSM)

The nonequilibrium two-site model (TSM) is based on the assumption that the insecticide compounds react with soil components at different rates and intensities [66,67,68,69]. TSM theoretically divides the soil medium into two domains, where the first domain of the sorption/desorption process is instantaneous, while the second one is time dependent. The sorption process described by TSM is shown using the following Equation (8):

(8) $$\frac{dq_{t}^{sor}}{dt}=k_{1}\left(q_{1eq}^{sor}-q_{1t}^{sor}\right)+k_{2}\left(q_{2eq}^{sor}-q_{2t}^{sor}\right)$$

where $q_{1t}^{sor}$ and $q_{2t}^{sor}$ (mg/kg) are the insecticide amounts sorbed on the two sites at given time *t*, while $k_{1}$ and $k_{2}$ are first-order rate constants (1/h). At equilibrium is $q_{eq}^{sor}=q_{1eq}^{sor}+q_{2eq}^{sor}$ and after integration Equation (8) becomes:

(9) $$q_{t}^{sor}=q_{1t}^{sor}\left(1-e^{-k_{1}t}\right)+q_{2t}^{sor}\left(1-e^{-k_{2}t}\right)$$

The desorption process of insecticides in soil according to the TSM is shown using Equation (10):

(10) $$-\frac{dq_{t}^{des}}{dt}=k_{1}\left(q_{1t}^{des}-q_{1eq}^{des}\right)+k_{2}\left(q_{2t}^{des}-q_{2eq}^{des}\right)$$

where $q_{1t}^{des}$ and $q_{2t}^{des}$ are amounts of insecticide on the suface of the soil at given time (mg/kg), while $k_{1}$ and $k_{2}$ are the first-order rate constants (1/h). Integrated form of Equation (10) is expressed as:

(11) $$q_{t}^{des}={q_{eq}^{des}+q}_{1t}^{des}e^{-k_{1}t}+q_{2t}^{des}e^{-k_{2}t}$$

where $q_{eq}^{des}$ is amount of pesticide sorbed at desorption equilibrium (mg/kg).

#### 4.6.3. Weber–Morris Intraparticle Diffusion Model (WMM)

WMM assumes that sorption/desorption processes depend on diffusion on/in the soil matrix (film, surface, and pore diffusion), representing a sorbent as well as convective diffusion in the solution containing the insecticide molecules. The sorption process is represented by Equation (12).

(12) $$q_{t}^{sor}=k_{id}^{sor}t^{1/2}+I$$

where $k_{id}^{sor}$ is the intraparticle diffusion rate constant (mg/(kg h^1/2^)), and *I* is a constant indicating the thickness of the boundary layer.

If the value of parameter $q_{t}^{sor}$ show linear dependence on $t^{1/2}$, and if the linear function passes through the origin, then intraparticle diffusion is involved in the sorption/desorption processes, and can have a significant impact on the rate of the overall process. Simultaneously, if the function does not pass through the origin, that is an indication of a certain degree of control of the boundary layer, i.e., intraparticle diffusion is not the only rate-limiting step of the sorption/desorption process, and therefore other kinetic processes can also control the rate of sorption/desorption, and take place at the same time.

The desorption process presented by WMM is expressed by Equation (13):

(13) $$q_{t}^{des}={-k}_{id}^{des}t^{1/2}-I$$

#### 4.6.4. Elovich’s Model (EM)

The Elovich’s model (EM) assumes that the kinetics of sorption/desorption processes takes place in two phases: the first, characterized by the rapid movement of the insecticide to the most accessible parts of the soil as sorbent while in the second, the slower reaction is particle diffusion of insecticide in/on the soil’s micropores. EM for sorption process can be expressed by Equation (14).

(14) $$\frac{dq_{t}^{sor}}{dt}={Xe}^{-Yq_{t}^{sor}}$$

The constant X is directly related to the rate of the first reaction phase of the sorption/desorption processes and indicating possible deviations of the rate change from the exponential law, while the constant Y predicts that the reaction rate follows first-order kinetics throughout the entire time.

At the boundary conditions $q_{t}^{sor}=0$ and *t* = 0 Equation (14) becomes:

(15) $$q_{t}^{sor}=\frac{1}{Y}lnt+\frac{1}{Y}ln(XY)$$

In the linear form of EM, [1/Y ln (XY)] and 1/Y represent the sorbed insecticide amount (mg/kg) during the fast phase (approximately until the 1. h of the reaction), and the amount in the slow sorption/desorption reaction, respectively.

The desorption process can be expressed by Equation (16):

(16) $$-\frac{dq_{t}^{des}}{dt}={Xe}^{-Yq_{t}^{des}}$$

Integrating Equation (16) at the boundary conditions $q_{t}^{des}$=$q_{eq}^{sor}$ and *t* = 0, the linear form of Equation (16) was obtained:

(17) $$q_{t}^{des}=q_{eq}^{sor}-\left(\frac{1}{Y}lnt+\frac{1}{Y}ln(XY)\right)$$

### 4.7. Statistical Analysis

All experimental data were presented as a mean of three determinations with standard deviation. Sorption/desorption experimental results were tested using the application of non-linear regression models available with the software Wolfram Research Mathematica^®^ V.12.0 (Wolfram Research Co., Champaign, IL, USA). As a measure of the goodness of fit of the experimental results and the results obtained by modelling, the following parameters were used: coefficient of multiple determination (*R*^2^), the scaled root mean squared error (SRMSE), and the minimum error level of *χ*^2^ test (err-%). The *χ*^2^ test can be used as the best parameter to test the goodness of fit, since it includes freedom degrees for each kinetic model at the desired level (5%) and compares the calculated *χ*^2^ value with the *χ*^2^ standard value [70]. A suitable model should pass the test if *χ*^2^ < *χ*^2^_tabulated_ at the chosen level of significance. The minimum error (err-%) at which the *χ*^2^ test is passed can be calculated by Equation (17):

(18) $$err(\%)=100x\sqrt{\frac{1}{{\chi^{2}}_{tab}}\sum_{i=1}^{N}\frac{{\left(M_{exp,i}-M_{pred,i}\right)}^{2}}{\overset{-}{{M_{exp,i}}^{2}}}}$$

The model with the smallest error (err-%) can be used as the most appropriate, since it describes the experimental data in the most robust way [70]. In Equation (18), *M*_exp_ and *M*_pred_ are the experimental and predicted data, $\overset{-}{M_{exp,i}}$ is the mean of all experimental data, N number of measurements, and *χ*^2^_tab_ is tabulated *χ*^2^ value for appropriate degrees of freedom at *p* = 0.05.

Descriptive statistics and all other statistical analyses were performed by the Statistica^®^ software V.14.0. (StatSoft, Inc., Tulsa, OK, USA) at a significance level of *p* < 0.05. The effect of physico-chemical soil characteristics on the insecticide sorption/desorption parameters obtained by mathematical modelling were tested by correlation matrix. Furthermore, to determine the mutual influence of soil characteristics and sorption/desorption parameters in the analysed soils, as well as similarities and correlations between variables, a factor analysis based on principal component analysis (PCA) was performed on the pooled analytical data.

## 5. Conclusions

Out of the four tested mathematical models (Lagergren’s pseudo first-order model, two-site model, Weber–Morris intraparticle diffusion model and Elovich’s model), the two-site model showed the best fit to our experimental data. The data showed that, even though the two insecticides are very similar in size and chemical structure, their sorption/desorption kinetic behaviours differ. Thiacloprid showed more rapid sorption compared to acetamiprid, and a higher proportion sorbed at equilibrium, in all soils. Intra-particle diffusion seemed to be a relevant process in acetamiprid sorption, but not for thiacloprid. Desorption results showed that acetamiprid is more easily and more thoroughly desorbed than thiacloprid, in all soils. These differences in the kinetic behaviour can be attributed to variations in molecular structure, namely, the presence of the sulphur-containing thiazolidine ring in thiacloprid, which leads to disparities in water solubility, acid–base properties, lipophilicity, and, consequently, the interactions with soil constituents.

## Figures and Tables

**Table 1:** Physico-chemical properties of the experimental soils.

Physico-Chemical Characteristics	Soil			
	S1	S2	S3	S4
Textural classes	clay loam	clay loam	clay loam	clay loam
pH ^(a)^	4.94 (±0.11)	5.29 (±0.06)	5.25 (±0.04)	5.55 (±0.04)
HA ^(b)^ (cmol/kg)	13.39 (±1.02)	4.62 (±0.46)	4.59 (±0.44)	6.59 (±0.26)
CEC ^(c)^ (cmol/kg)	60.76 (±4.26)	48.28 (±1.54)	49.76 (±1.91)	49.59 (±1.69)
Clay (%)	30.75 (±1.25)	35.26 (±0.86)	36.62 (±0.67)	37.60 (±1.07)
Ca^2+^ (mg/100 g)	38.9 (±0.6)	25.7 (±1.9)	20.4 (±3.9)	23.0 (±2.9)
Mg^2+^ (mg/100 g)	450.8 (±33.8)	401.1 (±21.6)	447.0 (±34.81)	352.4 (±24.4)
Na^+^ (mg/100 g)	23.4 (±57.2)	30.9 (±4.5)	28.5 (±8.7)	31.5 (±5.4)
K^+^ (mg/100 g)	286.7 (±32.9)	315.1 (±46.4)	240.8 (±29.1)	449.5 (±5.4)
humus (%)	2.64 (±0.03)	1.78 (±0.02)	2.01 (±0.34)	2.95 (±0.13)
TOC ^(d)^ (%)	2.59 (±0.10)	1.06 (±0.15)	1.71 (±0.01)	2.21 (±0.05)
C_oxHa_ ^(e)^ (%)	0.56 (±0.06)	0.42 (±0.06)	0.74 (±0.14)	0.47 (±0.10)
C_oxFa_ ^(f)^ (%)	1.06 (±0.08)	0.32 (±0.03)	0.10 (±0.01)	0.70 (±0.03)
N (%)	0.22 (±0.009)	0.13 (±0.002)	0.18 (±0.002)	0.22 (±0.011)
C (%)	2.13 (±0.014)	0.95 (±0.018)	1.28 (±0.005)	1.73 (±0.040)
H (%)	0.59 (±0.005)	0.37 (±0.005)	0.46 (±0.009)	0.49 (±0.014)
ratio C/H	3.58 (±0.02)	2.54 (±0.05)	2.81 (±0.07)	3.51 (±0.03)
ratio C/N	9.63 (±0.44)	7.39 (±0.05)	7.32 (±0.13)	7.71 (±0.23)
ratio 465/665	8.20 (±0.31)	5.45 (±0.30)	6.76 (±0.09)	7.19 (±0.15)

^(a)^ measured in soil + 0.01 M calcium chloride mixture (1:2.5 *w*/*V*); ^(b)^ hydrolitic acidity; ^(c)^ cation exchange capacity; ^(d)^ total organic carbon; ^(e)^ carbon of humic acids; ^(f)^ carbon of fulvic acids.

**Table 2:** The goodness of fit of the experimental results and the results obtained by modelling by pseudo first-order (PFOM), nonequlibrium two-site (TSM), Elovich (EM) and Weber–Morris (WMM) nonlinear kinetics models describing the sorption/desorption processes of acetamiprid in experimental soils represented by statistical indices: coefficient of multiple determination (*R*^2^), the scaled root mean squared error (SRMSE) and error of *χ*^2^ test.

Sorption					Desorption				
Statistical Parameter	S1	S2	S3	S4	Statistical Parameter	S1	S2	S3	S4
Pseudo First-Order (PFOM)									
*R* ^2 (a)^	0.9973	0.9981	0.9961	0.9976	*R* ^2 (a)^	0.9999	0.9995	0.9999	0.9999
SRMSE ^(b)^	0.0581	0.0463	0.0660	0.0518	SRMSE ^(b)^	0.0069	0.0215	0.0115	0.0112
err-% ^(c)^	4.67	3.71	5.30	4.16	err% ^(c)^	0.58	1.81	0.97	0.99
m ^(d)^	8 (*χ*^2^ _tab_ = 15.507 at *p* = 0.05)				m ^(d)^	7 (*χ*^2^ _tab_ = 14.067 at *p* = 0.05)			
Nonequlibrium Two-Site (TSM)									
*R* ^2 (a)^	0.9999	0.9999	0.9999	0.9999	*R* ^2 (a)^	0.9999	0.9999	0.9999	0.9999
SRMSE ^(b)^	0.0107	0.0123	0.0058	0.0120	SRMSE ^(b)^	0.0033	0.0077	0.0020	0.0019
err-% ^(c)^	0.96	1.10	0.51	1.07	err-% ^(c)^	0.31	0.73	0.19	0.18
m ^(d)^	6 (*χ*^2^ _tab_ = 12.592 at *p* = 0.05)				m ^(d)^	6 (*χ*^2^ _tab_ = 12.592 at *p* = 0.05)			
Elovich (EM)									
*R* ^2 (a)^	0.9880	0.9789	0.9534	0.9360	*R* ^2 (a)^	0.9050	0.9503	0.9449	0.9537
SRMSE ^(b)^	0.0437	0.0774	0.0967	0.1322	SRMSE ^(b)^	0.1268	0.0803	0.0888	0.0841
err-% ^(c)^	3.51	5.92	7.75	10.61	err-% ^(c)^	10.18	6.45	7.13	6.76
m ^(d)^	8 (*χ*^2^ _tab_ = 15.507 at *p* = 0.05)				m ^(d)^	8 (*χ*^2^ _tab_ = 15.507 at *p* = 0.05)			
Weber–Morris (WMM)									
*R* ^2 (a)^	0.9172	0.7143	0.7370	0.8626	*R* ^2 (a)^	0.6170	0.6819	0.9682	0.9576
SRMSE ^(b)^	0.1864	0.2956	0.2903	0.2055	SRMSE ^(b)^	0.3896	0.2986	0.0654	0.0765
err-% ^(c)^	14.97	23.73	23.31	16.51	err-% ^(c)^	32.68	24.03	5.96	7.88
m ^(d)^	8 (*χ*^2^ _tab_ = 15.507 at *p* = 0.05)				m ^(d)^	8 (*χ*^2^ _tab_ = 15.507 at *p* = 0.05)			

^(a)^—coefficient of multiple determination; ^(b)^— scaled root mean squared error; ^(c)^—minimum error level of *χ*^2^ test; ^(d)^—degrees of freedom = number of measurements—number of model parameters.

**Table 3:** The goodness of fit of the experimental results and the results obtained by modelling by pseudo first-order (PFOM), nonequlibrium two-Site (TSM), Elovich (EM) and Weber–Morris (WMM) nonlinear kinetics models describing the sorption/desorption processes of thiacloprid in experimental soils represented by statistical indices: coefficient of multiple determination (*R*^2^), the scaled root mean squared error (SRMSE) and error of *χ*^2^ test.

Sorption					Desorption				
Statistical Parameter	S1	S2	S3	S4	Statistical Parameter	S1	S2	S3	S4
Pseudo First-Order (PFOM)									
*R* ^2 (a)^	0.9997	0.9995	0.9989	0.9992	*R* ^2 (a)^	0.9999	0.9999	0.9999	0.9999
SRMSE ^(b)^	0.0191	0.0231	0.0348	0.0290	SRMSE ^(b)^	0.0012	0.0083	0.0076	0.0018
err-% ^(c)^	1.53	1.86	2.78	2.33	err-% ^(c)^	0.10	0.70	0.64	0.15
m ^(d)^	8 (*χ*^2^ _tab_ = 15.507 at *p* = 0.05)				m ^(d)^	7 (*χ*^2^ _tab_ = 14.067 at *p* = 0.05)			
Nonequlibrium Two-Site (TSM)									
*R* ^2 (a)^	0.9999	0.9999	0.9999	0.9999	*R* ^2 (a)^	0.9999	0.9999	0.9999	0.9999
SRMSE ^(b)^	0.0011	0.0131	0.0070	0.0066	SRMSE ^(b)^	0.0076	0.0017	0.0018	0.0007
err-% ^(c)^	0.10	1.17	0.62	0.59	err-% ^(c)^	0.72	0.16	0.17	0.06
m ^(d)^	6 (*χ*^2^ _tab_ = 12.592 at *p* = 0.05)				m ^(d)^	6 (*χ*^2^ _tab_ = 12.592 at *p* = 0.05)			
Elovich (EM)									
*R* ^2 (a)^	0.7225	0.6670	0.7156	0.8365	*R* ^2 (a)^	0.9030	0.9924	0.9911	0.9748
SRMSE ^(b)^	0.2572	0.2963	0.2668	0.2419	SRMSE ^(b)^	0.1333	0.0126	0.0169	0.0452
err-% ^(c)^	20.65	23.79	21.42	19.42	err-% ^(c)^	10.71	1.01	1.36	3.63
m ^(d)^	8 (*χ*^2^ _tab_ = 15.507 at *p* = 0.05)				m ^(d)^	8 (*χ*^2^ _tab_ = 15.507 at *p* = 0.05)			
Weber–Morris (WMM)									
*R* ^2 (a)^	0.7325	0.4635	0.5851	0.5524	*R* ^2 (a)^	0.5974	0.8860	0.7987	0.7477
SRMSE ^(b)^	0.3061	0.4025	0.3204	0.3312	SRMSE ^(b)^	0.3578	0.1682	0.2356	0.2576
err-% ^(c)^	24.58	35.31	29.36	30.11	err-% ^(c)^	30.12	14.78	18.56	20.46
m ^(d)^	8 (*χ*^2^ _tab_ = 15.507 at *p* = 0.05)				m ^(d)^	8 (*χ*^2^ _tab_ = 15.507 at *p* = 0.05)			

^(a)^—coefficient of multiple determination; ^(b)^— scaled root mean squared error; ^(c)^—minimum error level of *χ*^2^ test; ^(d)^—degrees of freedom = number of measurements—number of model parameters.

**Table 4:** Sorption and desorption parameters with statistical indices estimated by two-site (TSM) nonlinear kinetics model describing the sorption/desorption processes of acetamiprid and thiacloprid in experimental soils.

Sorption					Desorption				
Fitted/Statistical Parameter	S1	S2	S3	S4	Fitted/Statistical Parameter	S1	S2	S3	S4
Acetamiprid									
*k*_1_^sor (a)^ (1/h)	0.0452 (±0.0117)	14.1762 (±2.7099)	0.1355 (±0.0130)	0.3827 (±0.1377)	*k*_1_^des (c)^ (1/h)	1.6989 (±0.4831)	0.0715 (±0.0138)	1.7951 (±0.2964)	1.2687 (±0.2002)
*k*_2_^sor (b)^ (1/h)	11.2921 (±1.1083)	0.1135 (±0.0352)	12.4820 (±0.8839)	15.1937 (±0.4235)	*k*_2_^des (d)^ (1/h)	0.1326 (±0.0766)	1.5064 (±0.3155)	0.0259 (±0.0030)	0.0194 (±0.0031)
*q*_1_^sor (e)^ (mg/kg)	11.06 (±0.98)	48.96 (±0.64)	12.12 (±0.42)	11.56 (±1.27)	*q*_1_^des (g)^ (mg/kg)	2.34 (±0.78)	5.83 (±0.80)	3.07 (±0.23)	7.45 (±0.46)
*q*_2_^sor (f)^ (mg/kg)	66.44 (±0.62)	6.65 (±0.73)	56.32 (±0.38)	56.63 (±1.29)	*q*_2_^des (h)^ (mg/kg)	1.70 (±0.16)	8.30 (±0.91)	7.69 (±0.30)	3.25 (±0.22)
*R* ^2 (i)^	0.9999	0.9999	0.9999	0.9999	*R* ^2 (i)^	0.9999	0.9999	0.9999	0.9999
SRMSE ^(j)^	0.0107	0.0123	0.0058	0.0120	SRMSE ^(j)^	0.0033	0.0077	0.0020	0.0019
err-% ^(k)^	0.96	1.10	0.51	1.07	err-% ^(k)^	0.31	0.73	0.19	0.18
Thiacloprid									
*k*_1_^sor (a)^ (1/h)	1.2472 (±0.0886)	21.8250 (±0.0225)	1.1266 (±0.2358)	0.1877 (±0.0475)	*k*_1_^des (c)^ (1/h)	0.8057 (±0.0752)	0.0567 (±0.0067)	0.0888 (±0.0164)	0.1433 (±0.0266)
*k*_2_^sor (b)^ (1/h)	15.9947 (±0.8151)	2.4835 (±0.0235)	7.7060 (±0.9418)	8.9457 (±0.4233)	*k*_2_^des (d)^ (1/h)	0.0262 (±0.0041)	1.7781 (±0.3213)	2.2931 (±0.4723)	2.1306 (±0.7705)
*q*_1_^sor (e)^ (mg/kg)	11.13 (±0.63)	19.12 (±4.35)	24.89 (±4.74)	7.89 (±0.80)	*q*_1_^des (g)^ (mg/kg)	5.47 (±1.14)	5.36 (±0.23)	4.07 (±0.32)	1.91 (±0.20)
*q*_2_^sor (f)^ (mg/kg)	87.67 (±0.64)	55.57 (±4.30)	63.09 (±4.80)	85.61 (±0.77)	*q*_2_^des (h)^ (mg/kg)	0.27 (±0.01)	3.04 (±0.27)	3.58 (±0.37)	1.02 (±0.21)
*R* ^2 (i)^	0.9999	0.9999	0.9999	0.9999	*R* ^2 (i)^	0.9999	0.9999	0.9999	0.9999
SRMSE ^(j)^	0.0011	0.0131	0.0070	0.0066	SRMSE ^(j)^	0.0076	0.0017	0.0018	0.0007
err-% ^(k)^	0.10	1.17	0.62	0.59	err-% ^(k)^	0.72	0.16	0.17	0.06

^(a),(b),(c),(d),(e),(f),(g),(h)^ rate constants or sorbed/desorbed amount obtained by modelling with two-site model (TSM); ^(i)^ coefficient of multiple determination; ^(j)^ scaled root mean squared error; ^(k)^ minimum error level of *χ*^2^ test.

**Table 5:** Correlation coefficients between physico-chemical soil characteristics and acetamiprid/thiacloprid sorption parameters represented by correlation matrix (N = 1200; pooled data; 4 soils × 3 replication × 10 soil characteristics × 10 sorption parameters). Statistically significant correlations (*p* < 0.05) are presented with bold type numbers and with corresponding p values (written in parentheses in italics).

Variable	pH	HA ^(a)^	CEC ^(b)^	Clay	TOC ^(c)^	C_oxHa_ ^(d)^	C_oxFa_ ^(e)^	Ratio 465/665	Ratio C/H	Ratio C/N
Acetamiprid										
*q* ^sor (f)^	−0.472	0.809	0.816	−0.511	**0.950** (*p* = 0.049)	0.419	0.691	**0.994** (*p* = 0.006)	0.856	0.780
*k*_1_ (TSM) ^(g)^	0.106	−0.438	−0.450	0.061	−0.789	−0.613	−0.356	−0.853	−0.735	−0.391
*k*_2_ (TSM) ^(h)^	0.154	0.288	0.259	0.158	0.729	0.482	0.307	0.756	0.738	0.228
*q*_1_ (TSM) ^(i)^	0.098	−0.448	−0.455	0.063	−0.801	−0.591	−0.376	−0.860	−0.752	−0.400
*q*_2_ (TSM) ^(j)^	−0.234	0.576	0.587	−0.216	0.865	0.565	0.479	0.924	0.796	0.534
Thiacloprid										
*q* ^sor (f)^	−0.275	0.752	0.716	−0.367	**0.977** (*p* = 0.023)	0.302	0.723	**0.984** (*p* = 0.016)	0.937	0.708
*k*_1_ (TSM) ^(g)^	0.046	−0.412	−0.412	0.014	−0.786	−0.578	−0.356	−0.840	−0.747	−0.361
*k*_2_ (TSM) ^(h)^	−0.584	0.914	0.913	−0.665	**0.956** (*p* = 0.044)	0.287	0.797	**0.983** (*p* = 0.017)	0.857	0.894
*q*_1_ (TSM) ^(i)^	−0.137	−0.585	−0.405	0.212	−0.707	0.578	−0.861	−0.549	−0.838	−0.536
*q*_2_ (TSM) ^(j)^	−0.121	0.765	0.659	−0.343	**0.966** (*p* = 0.034)	−0.071	0.873	0.898	**0.993** (*p* = 0.001)	0.714

^(a)^ hydrolitic acidity; ^(b)^ cation exchange capacity; ^(c)^ total organic carbon; ^(d)^ carbon of humic acids; ^(e)^ carbon of fulvic acids; ^(f)^ sorbed amount in 96 h; ^(g),(h),(i),(j)^ parameters obtained by modelling with two-site model (TSM).

**Table 6:** Correlation coefficients between physico-chemical soil characteristics and acetamiprid/thiacloprid desorption parameters represented by correlation matrix (N = 1200 [pooled data; 4 soils × 3 replication × 10 soil characteristics × 10 desorption parameters]). Statistically significant correlations (*p* < 0.05) are presented with bold type numbers and with corresponding *p* values (written in parentheses in italics).

Variable	pH	HA ^(a)^	CEC ^(b)^	Clay	TOC ^(c)^	C_oxHa_ ^(d)^	C_oxFa_ ^(e)^	Ratio 465/665	Ratio C/H	Ratio C/N
Acetamiprid										
*q* ^des (f)^	0.423	−0.804	−0.798	0.481	**−0.965** (*p* = 0.035)	−0.378	−0.713	**−0.997** (*p* = 0.003)	−0.887	−0.771
*k*_1_ (TSM) ^(g)^	−0.302	0.445	0.512	−0.177	0.709	0.775	0.255	0.821	0.594	0.415
*k*_2_ (TSM) ^(h)^	0.023	−0.365	−0.372	−0.027	−0.750	−0.610	−0.303	−0.810	−0.710	−0.314
*q*_1_ (TSM) ^(i)^	0.896	−0.501	−0.671	0.685	−0.286	−0.695	−0.105	−0.451	−0.045	−0.541
*q*_2_ (TSM) ^(j)^	0.255	−0.859	−0.757	0.494	**−0.965** (*p* = 0.035)	0.139	−0.945	−0.894	**−0.981** (*p* = 0.019)	−0.818
Thiacloprid										
*q* ^des (f)^	0.114	−0.776	−0.656	0.366	**−0.946** (*p* = 0.048)	0.171	−0.908	−0.861	**−0.992** (*p* = 0.008)	−0.726
*k*_1_ (TSM) ^(g)^	−0.796	**0.990** (*p* = 0.010)	**0.997** (*p* = 0.003)	−0.913	0.805	0.0059	0.850	0.816	0.684	**0.996** (*p* = 0.004)
*k*_2_ (TSM) ^(h)^	0.838	−0.944	−0.949	**0.976** (*p* = 0.024)	−0.636	0.113	−0.813	−0.630	−0.519	**−0.965** (*p* = 0.035)
*q*_1_ (TSM) ^(i)^	−0.833	0.320	0.439	−0.750	−0.194	0.028	0.047	−0.119	−0.367	0.391
*q*_2_ (TSM) ^(j)^	0.242	−0.855	−0.732	0.532	−0.890	0.335	**−0.984** (*p* = 0.016)	−0.788	−0.928	−0.819

^(a)^ hydrolitic acidity; ^(b)^ cation exchange capacity; ^(c)^ total organic carbon; ^(d)^ carbon of humic acids; ^(e)^ carbon of fulvic acids; ^(f)^ desorbed amount in 96 h; ^(g),(h),(i),(j)^ parameters obtained by modelling with two-site model (TSM).

**Table 7:** Chemical structure and selected physico-chemical properties of acetamiprid and thiacloprid. Shaded areas designate the locations of the negative (orange/red) and positive (green/blue) charge [47].

Properties	Acetamiprid	Thiacloprid
Chemical structure	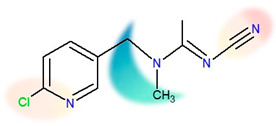	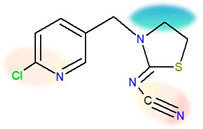
IUPAC name	N-[(6-chloropyridin-3-yl)methyl]-N’-cyano-N-methylethanimidamide	[3-[(6-chloropyridin-3-yl)methyl]-1.3-thiazolidin-2-ylidene]cyanamide
Molecular formula	C_10_H_11_ClN_4_	C_10_H_9_ClN_4_S
Molar mass/(g mol^−1^)	222.67	255.72
Melting point/°C	98.9	136.0
Vapor pressure/mPa	1.73·10^−4^ (20 °C)	3.00·10^−7^ (20 °C)
Water solubility/g L^−1^	2.95 (20 °C, pH 7)	0.19 (20 °C)
*K*_OW_	6.31	18.2
p*K*_a_	0.7	no dissociation
hydrogen bond donor count	0	0
hydrogen bond acceptor count	3	4
topological polar surface area/Å^2^	52.3	77.6

**Table 8:** Multiple reaction monitoring (MRM) transitions for quantification and confirmation of acetamiprid and thiacloprid and MS/MS conditions.

Compound	Q1 (m/z)	Q3 (m/z)	MRM Transition	CE (V)	DP (V)	EP (V)	CEP (V)
Acetamiprid	223.2	126.1	Quantitation	39.0	50.0	10.0	10.0
	223.2	99.1	Confirmation	67.0	50.0	10.0	10.0
Thiacloprid	253.1	126.1	Quantitation	29.0	50.0	10.0	10.0
	253.1	99.1	Confirmation	57.0	50.0	10.0	10.0

## Data Availability

Not applicable.

## Supplementary Materials

The following supporting information can be downloaded at: https://www.mdpi.com/article/10.3390/ijms24076548/s1.
