# Supplementary material for: Those That Remain: Sorption/Desorption Behaviour and Kinetics of the Neonicotinoids Still in Use
Source: Int J Mol Sci. 2023 Mar 31;24(7):6548. doi: 10.3390/ijms24076548 (PMC10095529; doi:10.3390/ijms24076548)
Supplement: Supplementary file 1 [file Table S1.pdf › Table S1.pdf]

Table S1. Sorption and desorption parameters with statistical indices estimated by Pseudo First-Order (PFOM) nonlinear kinetics models describing the sorption/desorption processes of acetamiprid and thiacloprid in experimental soils.

Sorption					Desorption				
Fitted/statistical parameter	S1	S2	S3	S4	Fitted/statistical parameter	S1	S2	S3	S4
Acetamiprid									
k^{sor} (a) (1/h)	7.8171 (±0.1587)	3.5941 (±0.0718)	6.7223 (±0.1031)	6.9781 (±0.1189)	k^{des} (b) (1/h)	0.9164 (± 0.0209)	0.4260 (± 0.0128)	0.0496 (±0.0150)	0.0492 (±0.0165)
q_{eq}^{sor} (c) (mg/kg)	71.43 (±1.42)	52.99 (±088)	64.17 (±1.41)	65.88 (±1.24)	q_{eq}^{des} (d) (mg/kg)	71.27 (±0.24)	43.62 (±0.65)	58.64 (±0.83)	60.37 (±0.87)
R^2 (e)	0.9973	0.9981	0.9961	0.9976	R^2 (e)	0.9999	0.9995	0.9999	0.9999
SRMSE (f)	0.0581	0.0463	0.0660	0.0518	SRMSE (f)	0.0069	0.0215	0.0115	0.0112
err-% (g)	4.67	3.71	5.30	4.16	err-% (g)	0.58	1.81	0.97	0.99
Thiacloprid									
k^{sor} (a) (1/h)	9.2870 (±0.8146)	3.3871 (±0.1635)	4.2017 (±0.3372)	7.0723 (±0.1242)	k^{des} (b) (1/h)	0.5634 (±0.0874)	0.1214 (±0.0316)	0.2613 (±0.0822)	0.2652 (±0.0522)
q_{eq}^{sor} (c) (mg/kg)	97.83 (±0.67)	74.35 (±0.63)	86.77 (±1.10)	91.15 (±0.96)	q_{eq}^{des} (d) (mg/kg)	96.46 (±0.06)	68.25 (±0.46)	81.33 (±0.41)	91.46 (±0.11)
SRMSE (f)	0.0191	0.0231	0.0348	0.0290	SRMSE (f)	0.0012	0.0083	0.0076	0.0018
err-%(g)	1.53	1.86	2.78	2.33	err-% (g)	0.10	0.70	0.64	0.15

(a). (b). (c). (d) – rate constants or sorbed/desorbed amount obtained by modelling with Pseudo-First Order (PFOM); (e) – coefficient of multiple determination (f) – Scaled Root Mean Squared Error; (g) – minimum error level of χ^2 test
