# Supplementary material for: Those That Remain: Sorption/Desorption Behaviour and Kinetics of the Neonicotinoids Still in Use
Source: Int J Mol Sci. 2023 Mar 31;24(7):6548. doi: 10.3390/ijms24076548 (PMC10095529; doi:10.3390/ijms24076548)
Supplement: Supplementary file 1 [file Table S2.pdf › Table S2.pdf]

Table S2. Sorption and desorption parameters with statistical indices estimated by Elowich (EM) nonlinear kinetics model describing the sorption/desorption processes of acetamiprid and thiacloprid in experimental soils.

Sorption					Desorption				
Fitted/statistical parameter	S1	S2	S3	S4	Fitted/statistical parameter	S1	S2	S3	S4
Acetamiprid									
$(1/Y)Ln(XY)^{sor}$ (a) (mg/kg)	65.95 (±2.21)	49.81 (±0.64)	58.33 (±1.21)	60.70 (±3.28)	$(1/Y)Ln(XY)^{des}$ (c) (mg/kg)	74.11 (±2.98)	50.23 (±2.41)	65.82 (±3.36)	67.14 (± 2.56)
$1/Y^{sor}$ (b)	2.43 (±0.18)	1.46 (±0.23)	2.58 (±0.64)	2.23 (±0.45)	$1/Y^{des}$ (d)	0.94 (±0.25)	1.93 (±0.52)	1.43 (±0.39)	1.36 (±0.18)
% (e)	85	90	85	87	% (e)	62	50	29	28
R^2 (f)	0.9880	0.9789	0.9534	0.9360	R^2 (f)	0.9050	0.9503	0.9449	0.9537
SRMSE (g)	0.0437	0.0774	0.0967	0.1322	SRMSE (g)	0.1268	0.0803	0.0888	0.0841
err-% (h)	3.51	5.92	7.75	10.61	err-% (h)	10.18	6.45	7.13	6.76
Thiacloprid									
$(1/Y)Ln(XY)^{sor}$ (a) (mg/kg)	94.25 (±4.14)	62.16 (±3.18)	75.44 (± 4.98)	85.08 (±3.95)	$(1/Y)Ln(XY)^{des}$(c) (mg/kg)	97.66 (±4.11)	73.39 (±2.32)	85.14 (±3.21)	93.11 (±5.16)
$1/Y^{sor}$ (b)	5.46 (±0.07)	4.17 (±0.38)	4.05 (±0.51)	4.46 (±0.35)	$1/Y^{des}$(d)	0.36 (±0.11)	1.26 (±0.23)	1.07 (±0.19)	0.46 (±0.13)
% (e)	97	93	91	93	% (e)	47	32	44	37
R^2 (f)	0.7225	0.6670	0.7156	0.8365	R^2 (f)	0.9030	0.9924	0.9911	0.9748
SRMSE (g)	0.2572	0.2963	0.2668	0.2419	SRMSE (g)	0.1333	0.0126	0.0169	0.0452
err-% (h)	20.65	23.79	21.42	19.42	err-% (h)	10.71	1.01	1.36	3.63

(a). (b). (c). (d) – sorbed/desorbed parameters obtained by modelling with Elowich (EM); (e) – amount sorbed/desorbed during the rapid phase (1 h) in regards to the sorbed/desorbed amount at 96 h; (f) – coefficient of multiple determination (g) – Scaled Root Mean Squared Error; (h) – minimum error level of χ^2 test
