# Supplementary material for: Those That Remain: Sorption/Desorption Behaviour and Kinetics of the Neonicotinoids Still in Use
Source: Int J Mol Sci. 2023 Mar 31;24(7):6548. doi: 10.3390/ijms24076548 (PMC10095529; doi:10.3390/ijms24076548)
Supplement: Supplementary file 1 [file Table S3.pdf › Table S3.pdf]

Table S3. Sorption and desorption parameters with statistical indices estimated by Weber-Morris (WMM) nonlinear kinetics model describing the sorption/desorption processes of acetamiprid and thiacloprid in experimental soils.

Sorption					Desorption				
Fitted/statistical parameter	S1	S2	S3	S4	Fitted/statistical parameter	S1	S2	S3	S4
Acetamiprid									
I^{sor} [(a)] (mg/kg)	64.65 (±5.72)	49.19 (±3.52)	57.44 (±6.12)	60.12 (±5.56)	I^{des} [(c)] (mg/kg)	74.32 (±0.33)	50.76 (±0.46)	66.74 (±0.28)	67.99 (±0.89)
k^{sor} [(b)] (mg/kg/$h^{1/2}$)	1.5228 (±0.0834)	0.8680 (±0.0346)	1.1771 (±0.3218)	1.2236 (±0.1235)	k^{des} [(d)] (mg/kg/$h^{1/2}$)	0.5048 (±0.0438)	1.0661 (±0.1348)	0.9409 (±0.0945)	0.8867 (±0.0825)
R^2 [(e)]	0.9172	0.7143	0.7370	0.8626	R^2 [(e)]	0.6170	0.6819	0.9682	0.9576
SRMSE [(f)]	0.1864	0.2956	0.2903	0.2055	SRMSE [(f)]	0.3896	0.2986	0.0654	0.0765
err-% [(g)]	14.97	23.73	23.31	16.51	err-% [(g)]	32.68	24.03	5.96	7.88
Thiacloprid									
I^{sor} [(a)] (mg/kg)	94.13 (±5.68)	62.05 (±7.83)	75.38 (±10.11)	84.60 (±9.21)	I^{des} [(c)] (mg/kg)	97.74 (±0.87)	74.02 (±1.26)	85.55 (±1.48)	93.26 (±0.85)
k^{sor} [(b)] (mg/kg/$h^{1/2}$)	0.6949 (±0.0935)	2.0069 (±0.2534)	1.9362 (±0.4587)	1.2941 (±0.1124)	k^{des} [(d)] (mg/kg/$h^{1/2}$)	0.1931 (±0.0745)	0.7762 (±0.1225)	0.6209 (±0.0978)	0.2605 (±0.0371)
R^2 [(e)]	0.7325	0.4635	0.5851	0.5524	R^2 [(e)]	0.5974	0.8860	0.7987	0.7477
SRMSE [(f)]	0.3061	0.4025	0.3204	0.3312	SRMSE [(f)]	0.3578	0.1682	0.2356	0.2576
err-% [(g)]	24.58	35.31	29.36	30.11	err-% [(g)]	30.12	14.78	18.56	20.46

[(a). (b). (c). (d)] – sorbed/desorbed parameters obtained by modelling with with Weber-Morris model (WMM);[(e)] – coefficient of multiple determination [(f)] – Scaled Root Mean Squared Error; [(g)] – minimum error level of χ^2 test
