# Supplementary material for: Those That Remain: Sorption/Desorption Behaviour and Kinetics of the Neonicotinoids Still in Use
Source: Int J Mol Sci. 2023 Mar 31;24(7):6548. doi: 10.3390/ijms24076548 (PMC10095529; doi:10.3390/ijms24076548)
Supplement: Supplementary file 1 [file ijms-24-06548-s001.zip › Table S4.pdf]

Table S4. Correlation coefficients between physico-chemical soil characteristics and acetamiprid/thiacloprid sorption parameters obtained by Pseudo First-Order (PFOM), Elowich (EM) and Weber-Morris (WMM) represented by correlation matrix (N = 1200; pooled data; 4 soils x 3 replication x 10 soil characteristics x 10 sorption parameters). Statistically significant correlations ($p < 0.05$) are presented with bold type numbers and with corresponding p values (written in parentheses in italics).

Variable	pH	HA[(a)]	CEC[(b)]	clay	TOC[(c)]	C_{oxHa}[(d)]	C_{oxFa}[(e)]	ratio 465/665	ratio C/H	ratio C/N
					**Acetamiprid**					
**q_{sor}[(f)]**	-0.472	0.809	0.816	-0.511	**0.950** (*p*=0.049)	0.419	0.691	**0.994** (*p*=0.006)	0.856	0.780
***k_1* (PFOM)** [(g)]	-0.270	0.643	0.644	-0.280	0.907	0.503	0.556	**0.954** (*p*=0.046)	0.840	0.601
**(1/Y)Ln(XY) (EM)** [(h)]	-0.397	0.803	0.789	-0.467	**0.973** (*p*=0.027)	0.351	0.727	**0.998** (*p*=0.002)	0.903	0.768
**(1/Y) (EM)** [(i)]	-0.244	0.375	0.444	-0.104	0.666	0.799	0.191	0.780	0.556	0.344
**C (WMM)** [(j)]	-0.364	0.790	0.770	-0.439	**0.975** (*p*=0.025)	0.341	0.725	**0.996** (*p*=0.004)	0.913	0.752
**k (WMM)** [(k)]	-0.528	0.866	0.868	-0.589	**0.959**(*p*=0.041)	0.354	0.748	**0.994** (*p*=0.006)	0.863	0.840
					**Thiacloprid**					
**q_{sor}[(f)]**	-0.275	0.752	0.716	-0.367	**0.977** (*p*=0.023)	0.302	0.723	**0.984** (*p*=0.016)	0.937	0.708
***k_1* (PFOM)** [(g)]	-0.394	0.919	0.845	-0.600	**0.978** (*p*=0.022)	-0.048	0.944	0.931	**0.956** (*p*=0.044)	0.886
**(1/Y)Ln(XY) (EM)** [(h)]	-0.348	0.835	0.791	-0.475	**0.997**(*p*=0.003)	0.204	0.812	**0.991** (*p*=0.009)	**0.955** (*p*=0.045)	0.796
**(1/Y) (EM)** [(i)]	0.060	-0.745	-0.613	0.330	-0.919	0.237	-0.906	-0.820	**-0.982** (*p*=0.018)	-0.694
**C (WMM)** [(j)]	-0.360	0.839	0.797	-0.483	**0.996** (*p*=0.004)	0.211	0.811	**0.992** (*p*=0.008)	**0.951** (*p*=0.049)	0.800
**k (WMM)** [(k)]	0.452	-0.948	-0.876	0.667	**-0.954** (*p*=0.046)	0.097	**-0.965** (*p*=0.035)	0.904	-0.926	-0.922

[(a)] – hydrolitic acidity; [(b)] – cation exchange capacity; [(c)] – total organic carbon; [(d)] – carbon of humic acids; [(e)] – carbon of fulvic acids; [(f)] – sorbed amount in 96. h; [(g). (h). (i). (j). (k).] – parameters obtained by modelling with Pseudo-First Order (PFOM), Elowich (EM) and Weber-Morris (WMM) models.
