# Supplementary material for: Those That Remain: Sorption/Desorption Behaviour and Kinetics of the Neonicotinoids Still in Use
Source: Int J Mol Sci. 2023 Mar 31;24(7):6548. doi: 10.3390/ijms24076548 (PMC10095529; doi:10.3390/ijms24076548)
Supplement: Supplementary file 1 [file ijms-24-06548-s001.zip › Table S5.pdf]

Table S5. Correlation coefficients between physico-chemical soil characteristics and acetamiprid/thiacloprid desorption parameters obtained by Pseudo First-Order (PFOM), Elowich (EM) and Weber-Morris (WMM) represented by correlation matrix (N = 1200; pooled data; 4 soils x 3 replication x 10 soil characteristics x 10 sorption parameters). Statistically significant correlations ($p < 0.05$) are presented with bold type numbers and with corresponding p values (written in parentheses in italics).

Variable	pH	HA[(a)]	CEC[(b)]	clay	TOC[(c)]	C_{oxHa}[(d)]	C_{oxFa}[(e)]	ratio 465/665	ratio C/H	ratio C/N
					**Acetamiprid**					
**q_{des}**[(f)]	0.423	-0.804	-0.798	0.481	**-0.965** (*p*=0.035)	-0.378	-0.713	**-0.997** (*p*=0.003)	-0.887	-0.771
***k_1* (PFOM)** [(g)]	-0.853	0.831	0.848	**-0.980** (*p*=0.020)	0.421	-0.214	0.693	0.413	0.303	0.868
**(1/Y)Ln(XY) (EM)** [(h)]	-0.364	0.725	0.728	-0.388	0.934	0.467	0.624	**0.980** (*p*=0.020)	0.855	0.688
**(1/Y) (EM)** [(i)]	0.497	-0.842	-0.844	0.552	**-0.959** (*p*=0.041)	-0.376	-0.729	**-0.996** (*p*=0.004)	-0.866	-0.815
**C (WMM)** [(j)]	-0.341	0.703	0.706	-0.3659	0.927	0.481	0.604	**0.974** (*p*=0.026)	0.850	0.665
**k (WMM)** [(k)]	0.698	**-0.977** (*p*=0.023)	**-0.977** (*p*=0.023)	0.804	-0.907	-0.169	-0.850	-0.924	-0.797	**-0.968** (*p*=0.032)
					**Thiacloprid**					
**q_{des}** [(f)]	0.114	-0.776	-0.656	0.366	**-0.946** (*p*=0.048)	0.171	-0.908	-0.861	**-0.992** (*p*=0.008)	-0.726
***k_1* (PFOM)** [(g)]	-0.711	**0.950** (*p*=0.049)	**0.967** (*p*=0.033)	-0.781	0.901	0.271	0.794	0.938	0.776	0.942
**(1/Y)Ln(XY) (EM)** [(h)]	-0.254	0.768	0.718	-0.373	**0.987** (*p*=0.013)	0.231	0.764	**0.980** (*p*=0.020)	**0.960** (*p*=0.040)	0.722
**(1/Y) (EM)** [(i)]	0.159	-0.792	-0.688	0.383	**-0.972** (*p*=0.028)	0.075	-0.890	-0.905	**-0.999** (*p*=0.001)	-0.743
**C (WMM)** [(j)]	-0.256	0.768	0.719	-0.374	**0.987** (*p*=0.013)	0.234	0.763	**0.980** (*p*=0.020)	**0.959** (*p*=0.041)	0.722
**k (WMM)** [(k)]	0.168	-0.788	-0.692	0.375	**-0.980** (*p*=0.020)	0.023	-0.872	-0.922	**-0.999** (*p*=0.001)	-0.739

[a] – hydrolitic acidity; [b] – cation exchange capacity; [c] – total organic carbon; [d] – carbon of humic acids; [e] – carbon of fulvic acids; [f] – desorbed amount in 96. h; [g]. [h]. [i]. [j]. [k] – parameters obtained by modelling with Pseudo-First Order (PFOM), Elowich (EM) and Weber-Morris (WMM) models.
